# Metal(loid)s in surface sediments of the middle and lower basins of the Usumacinta River (southeastern Mexico): variability and selenium enrichment

**DOI:** 10.7717/peerj.21776

**Published:** 2026-10-05

**Authors:** Javier Alcocer, Federico Páez-Osuna, Selene Olea-Olea, Luis A. Oseguera, Raúl A. Silva-Aguilera

**Affiliations:** 1 Grupo de Investigación en Limnología Tropical, FES Iztacala, Universidad Nacional Autónoma de México, Tlalnepantla, Estado de Mexico, Mexico; 2 Instituto de Ciencias del Mar y Limnología, Unidad Académica Mazatlán, Universidad Nacional Autónoma de México, Mazatlán, Sinaloa, Mexico; 3 Departamento de Dinámica Terrestre Superficial, Instituto de Geología, Universidad Nacional Autónoma de México, Ciudad de México, Ciudad de México, Mexico

**Keywords:** Heavy metals, Selenium, Enrichment factor, Geochemical index, Gulf of Mexico, Fluvial ecosystems

## Abstract

Large tropical rivers remain poorly characterized relative to the widely studied large temperate rivers. The Usumacinta River in southern Mexico is North America’s most extensive tropical fluvial system. Increasing anthropogenic pressures in the Usumacinta basin (*e.g.*, agriculture, cattle raising, forest fires, deforestation, and urban development) threaten ecosystem health. However, our understanding of the characteristics of the basins, especially on metal(loid) remains limited. To address this, we investigated metal(loid)s in sediments along the Usumacinta River. Thirty-four surface sediments from 17 stations within the Usumacinta’s mainstream and principal tributaries were sampled for metal(loid) analysis during the rainy and dry seasons. The sediment grain size and organic matter were analyzed and the concentrations of aluminium (Al), arsenic (As), barium (Ba), beryllium (Be), cadmium (Cd), chromium (Cr), cobalt (Co), copper (Cu), iron (Fe), lead (Pb), lithium (Li), magnesium (Mg), manganese (Mn), nickel (Ni), silver (Ag), selenium (Se), strontium (Sr), antimony (Sb) , thallium (Tl), tin (Sn), vanadium (V), and zinc (Zn) were quantified by Inductively Coupled Plasma Mass Spectrometry. The indices enrichment factor (EF), geo-accumulation index (Igeo), contamination factor (CF), and pollution load index (PLI) were used to assess the health status of metal(loid)s in the surface sediments of the middle and lower basins of the Usumacinta River. Metal(loid) concentrations in Usumacinta River sediments fall within or below the range reported for tropical rivers with comparable geological settings, suggesting limited anthropogenic metal contributions. While the EFs of Ag, Ba, Be, Cu, Sr, Tl, and V was <1.5, suggests that they are linked to weathering, those of As, Co, Mn, Pb, Sb, Sn, and Zn were 1.0 < EF < 10 in various cases, Se was consistently >10 in all stations, seasons, and in the middle and lower basins, evidencing that sediments are Se-enriched. Using four pollution indexes, we found that the sediments of the Usumacinta River are either uncontaminated or moderately contaminated (enriched) with As, Cd, Cu, Cr, Ni, Pb, Sb, V, and Zn. They are consistently enriched in Se from natural sources across both basins and seasons, but this enrichment may be modulated by an anthropogenic signal. In general, results showed that concentrations were higher during the dry than during the rainy season, and that concentrations in the lower basin were higher than in the middle basin. EFs of most elements and stations were <1.5, likely indicating that these metal(loid)s are derived from natural weathering; only the EFs of Se were >10 in all stations and seasons across both basins, suggesting sediment enrichment. This enrichment can be linked specifically to the Cretaceous marine sedimentary rocks or the occurrence of seleniferous in the Sierra de los Cuchumatanes. However, an additional contribution related to phosphate-based agricultural inputs may be added. The patterns thus indicate a superposition of a regional geogenic baseline and a seasonally modulated anthropogenic signal.

## Introduction

Rivers are water bodies and one of Earth’s most vital natural resources (Singh et al., 2017). They provide numerous essential ecological services, including water transport, aquaculture, diverse habitats for aquatic life, and support for biodiversity (Fang et al., 2019). Many activities, including mining, agriculture, industry, and urbanization, impact rivers and lead to the buildup of contaminants, such as metal(loid)s (Páez-Osuna et al., 2017; Páez-Osuna et al., 2023). For this reason, numerous studies have focused on these elements in rivers worldwide for decades (*e.g.*, Páez-Osuna et al., 1987; Gomes Ribeiro et al., 2017; Fang et al., 2019; Cuevas et al., 2023; Kim & Hong, 2023; Zhang et al., 2024; Ning et al., 2025; Li et al., 2025). Although metal(loid)s naturally occur in the Earth’s crust, human activities increase environmental levels of these metals. High metal concentrations are toxic, persistent, and can bioaccumulate (Páez-Osuna et al., 2017).

River sediments are an ecosystem component that provide nutrients for phototrophic organisms and serve as secondary sources of pollutants, including metal(loid)s (Fang et al., 2019). Metal(loid)s transported by rivers are easily adsorbed onto sediments. However, natural and human-made factors influence metal(loid) distribution, chemical speciation, and concentration in river sediments (Forstner & Wittmann, 1981; Horowitz, 1985; Páez-Osuna et al., 2017). The former includes rock weathering and soil erosion; the latter comprises sewage, industrial waste discharges, mining effluents, and agrochemical leaching (Fang et al., 2019). Rivers accumulate metal(loid)s as they flow through areas affected by both natural processes and human activities. The latter can alter the spatial distribution of metal(loid)s in sediments. Therefore, understanding how metal(loid)s are distributed in river sediments is crucial for risk assessment and the protection of aquatic ecosystems (Fang et al., 2019).

Large rivers from temperate and tropical regions have been extensively studied for metal(loid)s in China (*e.g.*, Wang et al., 2015; Fang et al., 2019; Deng et al., 2022; Zhang et al., 2024), India (*e.g.*, Jain, 2004; Singh et al., 2013; Singh et al., 2017), Korea (Kim & Hong, 2023), and Europe (Di Bella et al., 2024). However, research is limited in Africa (Ramadan et al., 2021) and in Central America and Mexico (Páez-Osuna, Botello & Villanueva, 1986). As a result, there is a knowledge gap regarding metals in the sediments of large tropical rivers in these regions. Tropical rivers supply more than 60% of the sediments discharged into the world’s oceans (Gaillardet, Dupré & Allègre, 1995). High temperatures, heavy rainfall, and abundant biological activity in these regions enhance chemical weathering of the basins (Lasaga et al., 1994).

Among the metal(loid)s transported by rivers, selenium (Se) exhibits particularly strong geogenic control, as its enrichment in fluvial and lacustrine sediments has frequently been linked to the weathering of organic-rich marine sedimentary rocks (Presser & Swain, 1990), including black shales and fine-grained carbonates, where Se is preferentially sequestered under reducing depositional conditions (Fordyce, 2013). These lithologies are widespread in many tropical regions characterized by intense chemical weathering, and their erosion can lead to downstream Se enrichment even in the absence of direct anthropogenic inputs. In Mesoamerica, large portions of the Maya Block are underlain by Mesozoic marine sedimentary successions, suggesting that the geological context may play a key role in controlling Se distributions in riverine sediments (Radmacher et al., 2021), alongside potential secondary contributions from agricultural practices. It is also important to acknowledge that pre-Columbian land use may have influenced the landscape and sediment dynamics; however, the intensity and spatial extent of these early anthropogenic activities were likely more limited than those of more recent, large-scale human pressures.

The main human activities in the Usumacinta basin are crop agriculture (4,800 km^2^), cattle raising (924 km^2^), and limited urban development (13.5 km^2^) (IGN, 2018), which could cause irreversible damage to this seemingly untouched environment. The Grijalva River, from a neighboring basin, was artificially diverted to join the Usumacinta River approximately 15 km upstream of its mouth into the Gulf of Mexico (Muñoz Salinas et al., 2016). Unlike the Usumacinta, the Grijalva River has been dammed at four sites (Angostura, Chicoasén, Malpaso, Peñitas) and polluted (Laino-Guanes et al., 2015) through agriculture, cattle raising, urban development, oil and gas field exploitation, and mining camps (SGM, 2020a; SGM, 2020b).

Despite its importance, the Usumacinta River has not yet been studied concerning metal(loid)s. Previous research on the Usumacinta River primarily focuses on the lower basin and coastal plains, including sediment load and transport (Muñoz Salinas et al., 2016), hydrological characterization, and nutrient levels (Cruz-Ramírez et al., 2019). The studies along the Mexican section of the Usumacinta River are recent and cover aspects of suspended sediments (Oseguera et al., 2018), water chemistry (Castillo et al., 2023; Olea-Olea et al., 2022), microbiology (Durán-Mendoza et al., 2018), and carbon dynamics (Cuevas-Lara et al., 2021; Soria-Reinoso et al., 2022).

This work provides information on metal(loid)s in sediments along the Usumacinta River, North America’s longest tropical river system. To do this, we examined three questions: What is the concentration of 22 metal(loid)s in the surface sediments of the Usumacinta River? How does the spatial distribution of metal(loid)s and seasonal changes (dry and rainy) vary? Is there evidence of enrichment and human sources of metal(loid)s in the Usumacinta River? Our hypotheses were that: (1) water-rock interactions modify the metal(loid) composition in the middle and lower basins of the Usumacinta River due to different weathering products; (2) tropical seasonality causes variations in metal(loid) concentrations; and (3) human activities mainly in the lower basin lead to higher levels of pollution-related metals such as cadmium (Cd), lead (Pb), and zinc (Zn).

## Materials and Methods

### Study area and sampling

In Mexico, the Usumacinta River basin spans two central geomorphological units—the middle and lower basins (Hudson et al., 2005). The middle basin includes a mountainous highland area that features the Sierra de los Cuchumatanes, the Sierra Madre de Chiapas, and the Altos de Chiapas. The Altos de Chiapas are mainly composed of Mesozoic marine and continental clastic carbonates with interbedded Cenozoic volcanic deposits (Morán-Zenteno et al., 1999). The Sierra de los Cuchumatanes primarily consists of Cretaceous limestones and dolomites (Marshall, 2007), while the Sierra Madre de Chiapas is characterized by dioritic and granitic rocks associated with a Paleozoic batholith (Morán-Zenteno et al., 1999).

The upper Usumacinta River basin drains the Sierra de los Cuchumatanes in northwestern Guatemala, a highland region of the Maya Block underlain predominantly by folded Paleozoic to Mesozoic sedimentary sequences (Walper, 1960; Fourcade et al., 1999). In adjacent northern Guatemala, well-characterized Cretaceous marine carbonate successions include the Cobán Formation (dolomitic, fossiliferous limestones) and the overlying Campur Formation (rudist-rich limestones) (Fourcade et al., 1999). These units record deposition on a shallow carbonate platform under warm marine conditions, and similar sequences have been interpreted to host organic-rich facies in transgressive settings along the Maya Block (Radmacher et al., 2021). Although these formations are not continuously exposed across the Cuchumatanes, they provide a suitable regional stratigraphic analog for the Cretaceous marine sedimentary rocks that contribute to the upper Usumacinta drainage. Direct measurements of Se in these units are scarce. Still, organic-rich marine carbonates and associated fine-grained facies are globally recognized as natural repositories of Se due to the preferential sequestration of Se under marine redox conditions (Fernández-Martínez & Charlet, 2009; Fordyce, 2013).

The Usumacinta River ranks second in discharge to the Gulf of Mexico after the Mississippi River. It measures approximately 1,100 km in length, has a drainage basin of about 112,550 km^2^, and a flow rate of around 2,678 m^3^ s^−1^ (Hudson et al., 2005). The river’s upper basin is in Guatemala, while the middle and lower basins are in Mexico (Fig. 1). It covers an area of 77,743.6 km^2^, situated between 89–92°W longitudes and 14–18°N latitudes (Benke & Cushing, 2009). The region’s climate is tropical and humid, with annual rainfall reaching up to 4,300 mm. The rainy season lasts from May to November, and the dry season is from December to April. The average yearly temperature ranges from 8–12 °C in the Altos Cuchumatanes (Guatemala) to 29–32 °C in the lower basin (Soares & García, 2017).

**Figure 1:**
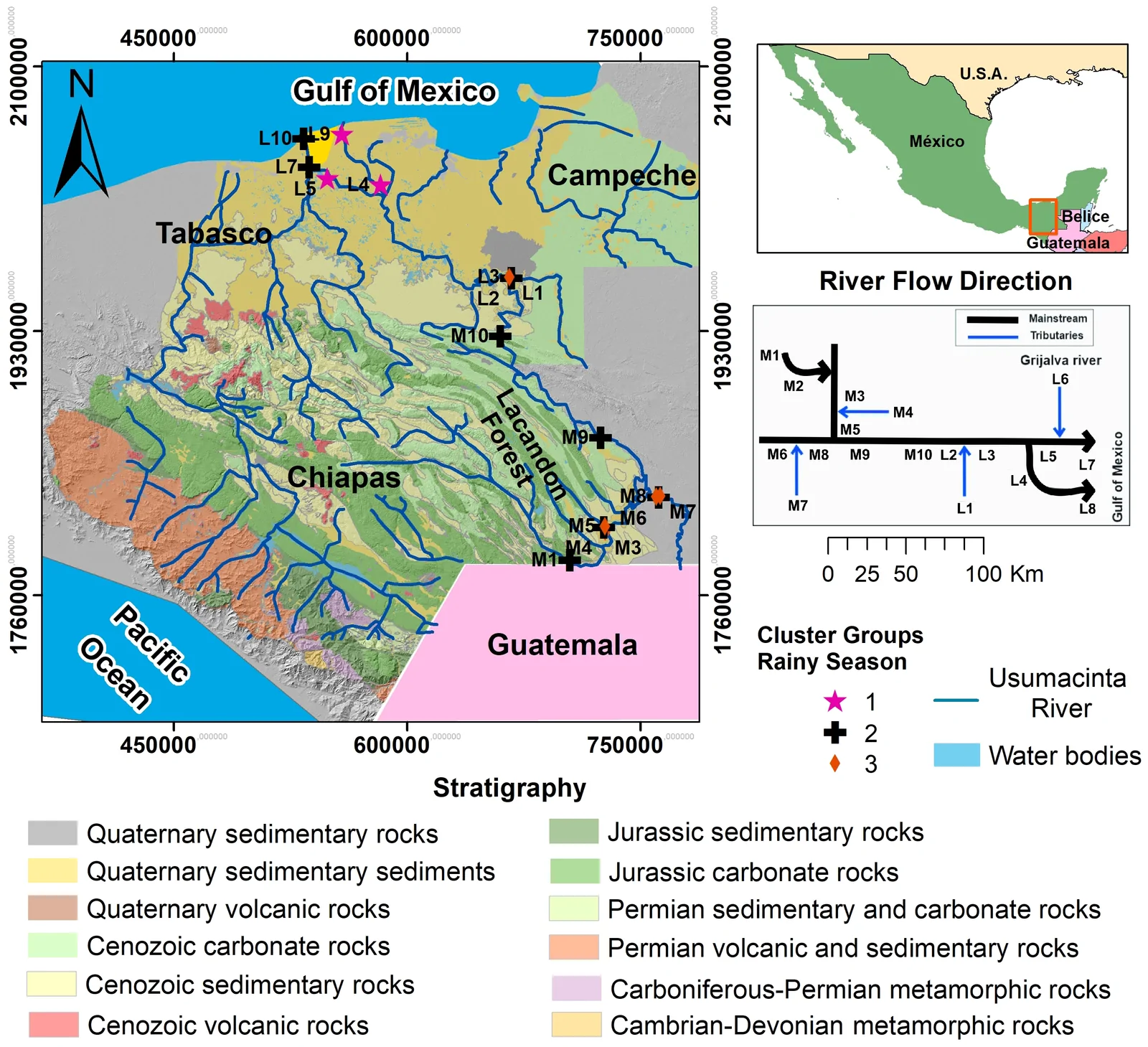
Geology of the Usumacinta River basin. Sampling stations are marked with L and M numbers, and a simplified river diagram is shown in the lower right corner (Geology from Servicio Geológico Mexicano SGM, references in the Supplementary Information).

The flora in the middle basin includes tropical and subtropical forests (Hudson et al., 2005). In the 2000s, evergreen, coniferous, and oak forests covered 85% of the middle basin. However, over the past 15 years, this area has shrunk by 14% (Peralta-Carreta et al., 2019). Conversion of native vegetation to agricultural land and grazing areas, and to urban development in the middle basin, has led to landslides and flooding during extreme rainfall events (García & Kauffer, 2011). In the lower portion of the basin, mangrove forests and various hydrophytes make up 8% of the Usumacinta-Grijalva basin (Sánchez-Hernández et al., 2013). Additionally, submerged and floating macrophytes reside in the lakes and floodplains connected to the main channel of the Usumacinta (Castillo, 2020).

Although a large portion of the Usumacinta Basin is protected (33%), about one-third of its surface is affected by deforestation, and half remains minimally disturbed (Cruz-Paz et al., 2018). The Usumacinta River basin is home to 3,803,869 residents (Pohlenz de Tavira, 2019). The most important cities include Cobán (Guatemala; 215,796 residents), Ocosingo (218,893 residents), San Cristóbal de Las Casas (209,591 residents), and Ciudad del Carmen (Mexico; 248,303 residents). The most significant urban developments in the Usumacinta River basin in Mexico include Tenosique (62,310 residents), Balancán (58,524 residents), and Centla (107,731 residents).

The destruction of jungle areas, driven by natural and human-caused burning, and the use of agrochemicals on land that was once jungle but is now unsuitable for crops and livestock, threatens the resilience of the basin’s ecosystems. This includes monocultures of oil palm, teak, eucalyptus, and other commercial species (Soares & García, 2017).

Although a detailed inventory of sanitary landfills and wastewater treatment plants in the Usumacinta River basin is unavailable, the need to develop and enhance sanitation infrastructure in the region is clear, as evidenced by the microbiological quality of the river water (Durán-Mendoza et al., 2018). This is underscored by the microbiological quality of the river water, as the Usumacinta River exhibits impairment characterized by elevated total and fecal coliform concentrations and the presence of pathogenic enterobacteria (*e.g.*, *Escherichia* and *Salmonella*). These conditions indicate fecal contamination and pose potential risks to aquatic biota and human health (Durán-Mendoza et al., 2018).

Sampling was carried out in the middle and lower basins of the Usumacinta River, with nine locations along the main channel (four in the middle basin and five in the lower basin), six in the main tributaries (five in the middle basin and one in the lower basin), and two in the distributaries, both located in the lower basin, totaling 17 sampling stations (Fig. 1, Table 1). A total of thirty-four sediment samples were collected from the mainstream and primary tributaries of the Usumacinta River; nine samples were taken in the middle basin and eight in the lower basin (Fig. 1) during each season: rainy (October–November 2017) and dry (April 2018). The samples were collected with an acrylic corer (10 cm diameter). After sampling, the top one cm was stored in polyethylene bags at 4 °C. The samples were freeze-dried (72 h, −55 °C, and 75 ×10^−3^ mbar) to remove water from the sediments and were homogenized prior to analysis.

**Table 1:** Sampling sites in the Usumacinta River’s middle (M) and lower (L) basins, with their altitude and geographical coordinates.

**Station**	**Code**	**Latitude** **UTM**	**Longitude** **UTM**	**Altitude** **(m asl)**	**Order**
Ixcan	M1	1782640	704727	158	Tributary
Lacantun	M3	1800790	727433	137	Tributary
Tzendales	M4	1803793	726631	134	Tributary
Lacantun-Tzendales	M5	1804437	727316	133	Tributary
Chixoy	M6	1823051	761812	107	Mainstream
Pasión	M7	1823953	762775	107	Tributary
Chixoy-Pasion	M8	1823414	760335	107	Mainstream
Frontera Corozal	M9	1861389	724442	95	Mainstream
Boca de Cerro	M10	1926693	660039	25	Mainstream
San Pedro	L1	1964679	667707	5	Tributary
Balancán	L2	1963988	666942	6	Mainstream
San Pedro-Balancán	L3	1964602	665786	5	Mainstream
Jonuta	L4	2023798	583080	2	Distributary
Usumacinta	L5	2028076	548738	1	Mainstream
Usumacinta-Grijalva	L7	2035408	537013	0	Mainstream
Arroyo Hondo	L9	2056374	557978	0	Distributary
Desembocadura	L10	2053519	533555	0	Mainstream

### Sediment grain size and chemical analyses

Sediment grain size was analyzed by dry sieving for gravels and sands, and by a Beckman Coulter LS230 Laser Diffraction Particle Analyzer for silts and clays (Folk, 1980). Sediment organic matter (SOM) was estimated by ignition at 550 °C (Ontiveros-Cuadras et al., 2014). The sediment metal(loid) analyses were performed at the Geochemical and Environmental Research Group of Texas A&M University. Concentrations of aluminium (Al), barium (Ba), beryllium (Be), Cd, chromium (Cr), cobalt (Co), copper (Cu), iron (Fe), Pb, lithium (Li), magnesium (Mg), manganese (Mn), nickel (Ni), silver (Ag), strontium (Sr), thallium (Tl), tin (Sn), vanadium (V), and Zn, along with the metal(loid)s antimony (Sb), arsenic (As), and Se, were measured by Inductively Coupled Plasma Mass Spectrometry (ICP-MS) (Agilent 7500ce, Santa Clara, CA, USA) after microwave digestion (CEM Corp., MARS-Xpress; Matthews, NC, USA) of aliquots of sediment samples with concentrated HCl + HF + HNO_3_ (Merck KGaA, Darmstadt, Germany) (United States Environmental Protection Agency, 2007). Replicates (*n* = 5) of the certified materials IAEA-158, IAEA-433, and NBS-1646 showed a recovery of 90–117% and a precision below 10% for most elements (Supplementary material Tables S0a–S0c). Two blanks were used for each batch during each digestion run (24 samples) to test for contamination; they were consistently clean.

### Pollution and geochemical indices

This study used four indices to assess the health status of metal(loid)s in the surface sediments of the middle and lower basins of the Usumacinta River: enrichment factor (EF), geo-accumulation index (Igeo), contamination factor (CF), and pollution load index (PLI). These four indices complement one another, particularly the first two; their synergy enables the identification of enrichment trends and concentration anomalies, thereby underscoring their importance and their widespread use. The EF values were calculated using the formula (Soto-Jimenez & Páez-Osuna, 2001): EF =(M/Al)_sample_/(M/Al)_crust_. The values for the Earth’s crust used (Martin & Meybeck, 1979) represent the average composition of surface rocks exposed to weathering. The normalizer was selected based on the highest correlation among potential candidates, considering granulometry and organic matter, despite the modest strength of the relationship (R^2^ ≈ 0.3). More details are available in the Supplementary Information (Fig. S2).

The Igeo was calculated using the equation Igeo = log_2_ [*Cn*/1.5*Bn* ], where *Cn* is the concentration of the element in the sediment, *Bn* is the background concentration of the metal(loid) (average crust concentration; Taylor, 1964), and 1.5 is the correction factor to account for background data (correction factor) due to the lithogenic effect (Taylor, 1964). The Igeo factor comprises seven classes (0–6), suggests increasing levels of metal enrichment relative to average crust, from unpolluted to highly or very highly polluted sediment, as detailed in the Supplementary Information.

The CF was calculated using the equation CF = (mean metal(loid) concentration at the contaminated site)/(metal(loid) average shale concentration), which Håkanson (1980) classified into four categories described in the supplementary information. Contamination degree (CD) is typically defined for river sediments as the sum of CF values from a specific sampling site, and it is categorized into four sediment contamination levels (Ahdy & Khaled, 2009, Supplementary Information).

The PLI was calculated for each site using the method described by Tomlinson et al. (1980), with the formula (for a site): PLI = [(CF1 × CF2 × CF3 × CF4 × …CF*n*)]^1/*n*^, where CF represents the contamination factor, and *n* is the number of parameters. The equation for each zone or basin is PLI = [site 1 × site 2 × site 3 …× site *n*]^1/*n*^, and the PLI for rivers is PLI = [zone 1 × zone 2 × zone 3 × zone *n*]^1/*n*^, where *n* is the number of contamination factors, sites, and zones. A PLI of 0 suggests no pollution; a PLI of 1 suggests baseline levels of pollutants; and a PLI greater than 1 likely indicates progressive deterioration of sites, zones (basin), or the river (Mohiuddin et al., 2010).

### Data treatment

Average concentrations were calculated for both basins during the dry and rainy seasons to determine if metal(loid) concentrations change across space and seasons. Data normality was tested using the Shapiro–Wilk test. Two-tailed statistical tests were performed comparing (a) dry season *versus* rainy season, (b) the middle basin in the dry season *versus* the lower basin in the dry season, and (c) the middle basin in the rainy season *versus* the lower basin in the rainy season. A Student’s *t*-test was used for normally distributed trace metal values, while the non-parametric *U* Wilcoxon-Mann–Whitney (Wilcoxon, 1992) tests were applied for non-normal distributions. A 0.05 confidence level was considered, and the hypotheses for both were: HA: mD*a* ≠ mD*b*; H0: mD*a* = mD*b*. D refers to the data array, and m to the corresponding mean. HA is the alternative hypothesis, which states that the means of data array *a* differ from those of data array *b*; H0 is the null hypothesis, and the means of data array *a* do not differ from those of data array *b*.

***Multivariate analysis*.** According to the statistical tests, eight metals (*i.e.,* Be, Cu, Fe, Li, Mg, Mn, Ni, Sr) showed no significant differences (*p* < 0.05) between seasons and basins. Therefore, further multivariate analyses were conducted on the remaining 14 elements (Al, As, Ag, Cd, Co, Cr, Ba, Pb, Sb, Se, Sn, Tl, V, and Zn). Principal Component Analysis (PCA) was used as an exploratory tool to evaluate patterns of co-variability among metal(loid)s and to support interpretations derived from univariate and spatial analyses. The data processing and statistical analysis were performed using R (R Core Team, 2021). Additionally, a hierarchical cluster analysis (Köhn & Hubert, 2014) was conducted in R using Ward’s method on the selected 14 trace metals across the dry and rainy seasons. This analysis aimed to group the sample sites based on similar trace metal compositions.

## Results

### Grain size and distribution of metal(loid)s in sediments

The mean grain size of sediment samples at each site in the lower basin (Fig. S1) predominantly ranged from 50 to 2,830 µm, with an average of 510 ± 700 µm. 75.5% of sites contained sand, 17.6% coarse silt, and 5.9% gravel. The middle basin exhibited coarser sediments (average: 840 ± 400 µm) compared to the lower basin (average: 140 ± 130 µm). The median grain size of the sediment in both basins differed significantly (*p* = 0.004). Additionally, the SOM content ranged from 0.7% to 7.9%, with a mean of 3.2 ± 2.0% (Fig. S1). The SOM in the middle basin was significantly lower (*p* = 0.010), averaging 2.1 ± 1.4%, compared to the lower basin’s average of 4.4 ± 2.1%.

The spatial distribution of metal(loid)s in the sediments of the Usumacinta River during the dry and rainy seasons is shown in the Supplementary Information (Fig. S2 and Table S2). During the dry season, most metal(loid)s increased from the middle to lower basins, except for Cr, Mn, and Ni, which decreased, while Co remained similar. During the rainy season, most elements increased in the middle and lower basins, except for Cu, which decreased. A general trend of increasing metal(loid) concentrations from the middle to the lower basin was evident for most elements in both seasons, especially during the rainy season. This pattern likely reflects not only the transport of weathered material from the upper basin but also its accumulation, potentially enhanced by increased SOM across this area.

Metal(loid) contents in the two basins and seasons varied widely. Except Cr (at the M5 site during the dry season) and Ni (at 15 sites during both seasons), these ranges did not exceed the PEL values (probable effect level in µg g^−1^) for As (17.0), Cd (3.5), Cr (90.0), Cu (197), Ni (36.0), Pb (91.3), and Zn (315), which mark the lower limit of the chemical concentration ranges typically or always linked with biological effects (Buchman, 2008; CCME, 2023).

Although regional comparisons offer a superficial way to examine sediment concentration levels across different areas by excluding various variables (such as grain size and organic matter), they still allow generalizing trends. The trace metal(loid) levels in the Usumacinta River sediments are lower or similar to those reported in other tropical rivers with comparable geology (Table 2). From this strong contrast, it can be generalized that the trace metal concentrations in the Usumacinta River sediments are below (As, Pb, Sb, V) or in the lower range (Al, Ba, Cd, Co, Cr, Zn) of other tropical rivers, with or without anthropic pollution, suggesting the absence or low presence of anthropogenic metals in the Usumacinta River.

**Table 2:** Average trace metal(loid) concentration (µg g^−1^ dw) in surface sediments from the Usumacinta River and large tropical rivers worldwide (– not analyzed; * with evidence of anthropogenic sources; values in parenthesis correspond to levels in the rainy season).

**River**	**As**	**Cd**	**Co**	**Cr**	**Cu**	**Ni**	**Pb**	**Zn**	**Reference**
Yangtze, China	–	1.0	–	108	60.0	41.9	49.2	230	Wang et al. (2011)
Yangtze, China	9.1*	0.19	–	79	60.0	41.9	23.8	29	Wang et al. (2015)
Pearl, China	–	–	19.5*	119*	–	–	73	199*	Liang et al. (2023)
Mekong, Asia	19*	–	–	183*	–	–	37*	83*	Fu et al. (2012)
Euphrates, Iraq	–	1.9	–	58.4	18.9	67.1	22.6	48	Salah, Zaidan & Al-Rawi (2012)
Ganges, India	–	0.64	20	158	56	51	20	67.8	Singh et al. (2013)
Ganges, Bangladesh	0.7*	1.9*	–	9.3*	–	–	6.4*	16*	Sayed Jewel et al. (2020)
Yamuna, India	–	10.1	–	–	22	–	58	62	Jain (2004)
Tapti, India	–	–	–	–	0.5–4.1	–	–	1.2–6.1	Marathe et al. (2011)
Kaveri, India	–	1.3	–	38.9	11.2	27.7	4.3	93.1	Raju, Somashekar & Prakash (2012)
Ghaghara, India	–	0.25	14.9	74.5	7.25	20.4	12.5	15.4	Singh et al. (2017)
Congo, R. Congo	1.7	0.16	5.8	43*	–	–	46.1*	394*	Suami et al. (2020)
Niger, Delta	–	1.4 (1.5)*	7.7 (8.5)	35 (41)*	–	–	42 (44)*	18(25)	Iwegbue et al. (2018)
Amazon, Brazil	1.2	–	–	65	37.5	26.7	83	110	Siqueira (2003)
Xingu, Brazil	0.6 (5.3)	0.80	–	23 (193)	–	–	11 (105)	426	Gomes Ribeiro et al. (2017)
Coatzacoalcos, Mexico	–	1.4*	23.3*	79.5*	35.7*	42.0*	36.3*	89.1*	Páez-Osuna, Botello & Villanueva (1986)
Usumacinta, Mexico	2.2 (3.7)	0.2 (0.5)	5.3 (6.7)	44 (60)	3.4 (3.6)	49.2 (66.7)	8.1 (5.4)	55 (52)	This study

## Discussion

The Usumacinta River’s main hydrological and physicochemical properties vary between the middle and lower basins and across rainy and dry seasons (Supplementary Information (Table S1)). During the dry season, the middle basin shows higher water discharge and dissolved oxygen levels but lower maximum depth, temperature, and electrical conductivity (*i.e.,* saline wedge closest to the sea stations) than the lower basin. For its part, the pH remains similar in both basins. In the rainy season, the middle basin exhibits higher water discharge, maximum depth, dissolved oxygen, and pH than the lower basin. Water temperature is higher in the lower basin, while electrical conductivity was similar in both basins (Cuevas-Lara et al., 2021).

### Normalization of the metal(loid) levels and identification of outliers

A significant correlation (*p* < 0.01) between reference metals (such as Al) and the grain-size parameter (mz) suggests that these metal(loid)s were important components of various significant fine-grained metal(loid) carriers and reveal a granular variability in the river sediments (Supplementary Information (Fig. S3)). Most analyzed metal(loid)s in sediments from the Usumacinta River showed a significant linear correlation with Al (Fig. 2); *R*-values ranged from 0.614 to 0.997. Silver, Cd, and Co were significantly correlated only when the sites were separated by season. Chromium showed no significant correlation (*p* > 0.05), while Sn exhibited an unexpected negative correlation (*r* =  − 0.940, *p* < 0.05). The latter suggests that as the sediments’ aluminates (and grain size) decrease, the Sn content increases, which is unusual, implying that another component in the sediments influences the variability of this metal.

**Figure 2:**
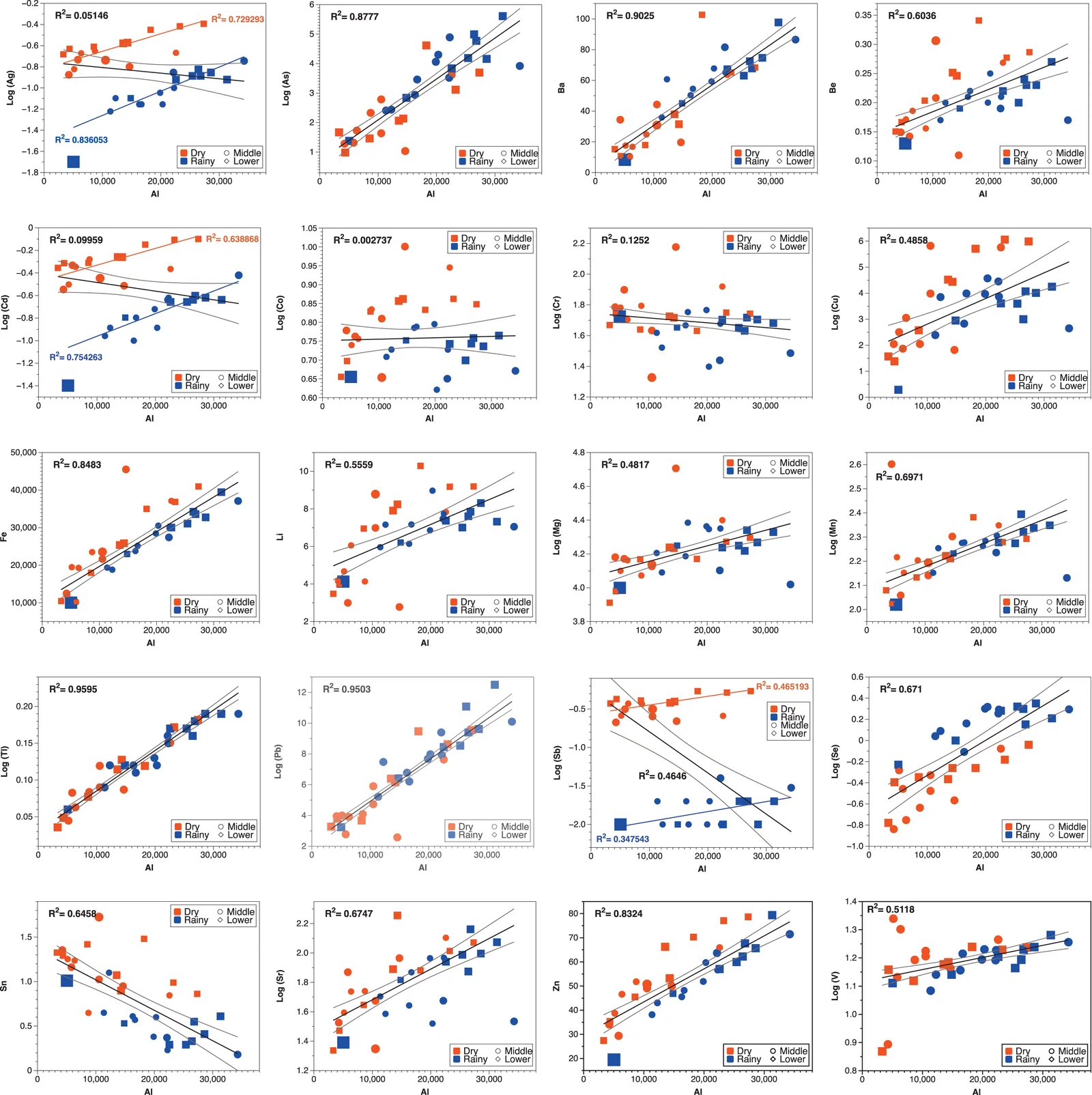
Relationships betweent metal(loid) and Al concentrations in sediments of the Usumacinta River. Scatter plots showing the relationships between metal(loid) and Al concentrations (µg g^−1^) in surface sediments of the Usumacinta River. The solid line indicates the regression line for the selected data, while the thin lines show the 95% prediction limits. In the Al/Ag, Al/Cd, and Al/Sb graphs, the orange line indicates the regression line for the dry season and the blue line the regression line for the rainy season. Small symbols represent points outside the prediction limits that were removed; large symbols represent points included in the final regression model. Except for the Log of concentration, both *X* and *Y* axes are measured in µg g^−1^.

Chromium and Sn variability may result from mineralization where aluminates are not involved. Although ultramafic rocks and soils vary in character and composition, they share the common feature of being enriched in certain elements, and three metals in particular form an ultramafic triad: Ni, Cr, and Co (Kierczak, Pietranik & Pedziwiatr, 2021). Therefore, at least these three metals can be linked to the ultramafic component of the volcanic pile. Correlations with Al showed that most elements are naturally occurring and mutually correlated. Exceptions include Sn, Sb, Cr, and Co; when Co and Sb are analyzed separately by season, they exhibit significant correlations. The behavior of Cr and Sn may also be influenced by the presence of minerals (or contaminants) with varying metal concentrations.

The metal(oid) level outliers—above the correlation lines (*i.e.,* highest concentration)—regarding Al (Fig. 2) were found at 11 sampling stations during the dry season, while only four were observed during the rainy season, suggesting that the flushing effect during heavy rains tends to homogenize the river. During the dry season, most outliers were located in M1 (five metals), M5 (four metals), L4 (six metals), L5 (four metals), and L9 (eight metals), whereas in the rainy season, outliers appeared in M6 (four metals) and M7 (two metals). The most common metal outliers during the dry season were Cu and Zn, while Pb was an outlier in the rainy season. Agriculture in the Lacantún River (middle basin) could be related to the higher concentrations of M1 (Cu, Sn, Tl) and M5 (Cr, Fe, Ni) during the dry season and L4 (Ag, Cd, Co, Cu, Sb, Zn), L5 (Ag, Cd, Co, Sb), and L9 (As, Ba, Cu, Pb, Sn, Zn) in the lower basin. This partly aligns with the presence of certain metals enriched in agrochemicals, as agriculture has been linked to Cd, Co, Cu, Ni, and Zn, mainly associated with fertilizers and fungicides (Páez-Osuna et al., 2017). Conversely, the greater abundance of igneous and metamorphic rocks in the basins of the Chixoy and La Pasión Rivers (Fig. 1) could explain the elevated concentrations in M6 (Ba, Pb) and M7 (As, Pb) during the rainy season. Therefore, these outliers or “concentration anomalies” for Cd, Co, Cu, and Zn seem to be linked to agrochemicals, while As, Ba, Pb, and probably Sn, Sb, Tl, and Ag appear to be connected to rock weathering in the region. While phosphate fertilizers are known to be generally enriched with As, Cd, and numerous metalloids, and fungicides, depending on their use, have a high content of Cu and Zn, the former are characterized by their highly variable concentration (Uday Kumar et al., 2023), which is related to the composition of the phosphate rock deposits from which the fertilizer is produced (Mortvedt, 1995). Phosphorite and apatite rocks used in phosphate fertilizer production contain several other minerals, including Cd and other elements. The amount of Cd (and other elements) incorporated in phosphate fertilizers depends on the type of phosphorite and apatite rocks (with low or high Cd content) (Suciu et al., 2022). Therefore, it is difficult to definitively link the different metal groups to agricultural activity with sufficient certainty.

### Assessment of metal(loid) enrichment

Many studies have assessed metal(loid) enrichment in river sediments using the EF and Igeo indices, but fewer have used other indices, such as CF and PLI (Fang et al., 2019; Fang et al., 2021, and references therein). The following subsections describe how EF, Igeo, CF, and PLI are applied in this study.

***Assessment by the EF index*.** The EF of all analyzed samples is shown in Fig. 3. The EF is primarily used to determine whether metal(loid)s originate from natural rock weathering or human-related pollution, helping to assess environmental contamination (Liang et al., 2023). The EF of various metal(loid)s (Ag, Ba, Be, Cu, Sr, Tl, and V) in this study were found to be <1.5 (Fig. 3), suggesting that these were not enriched or were only slightly enriched and, therefore, may have entirely originated from natural weathering processes. Conversely, the average EFs of As, Co, Mn, Pb, Sb, Sn, and Zn across sites ranged from 1.0 to 10 (Fig. 3), suggesting that sediments are either low or moderately enriched (potentially contaminated). However, the carbonate dilution effect cannot be ignored in these cases, as carbonates in the study area can be a significant component, attenuating EF values.

**Figure 3:**
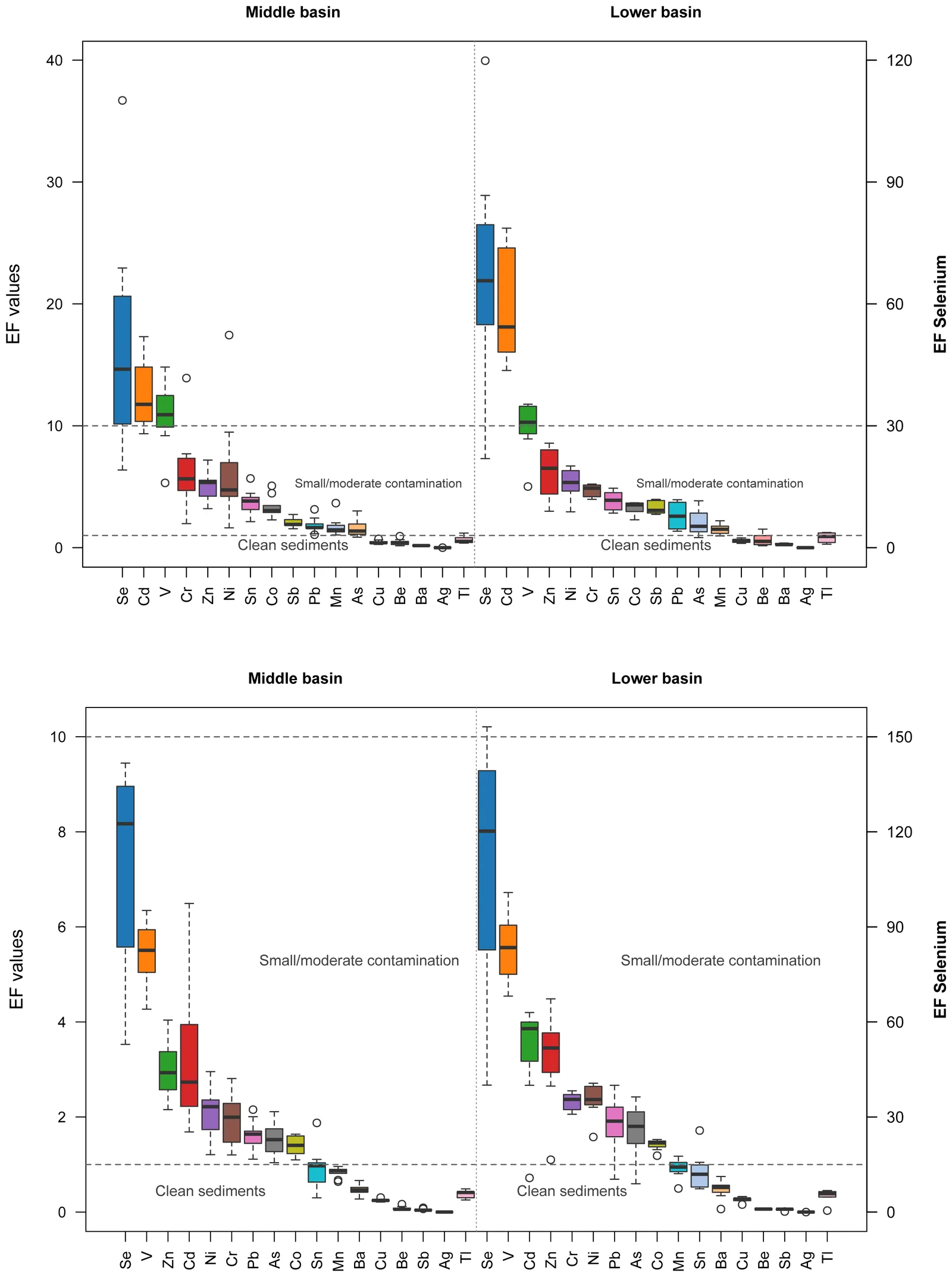
Enrichment factors (EFs) for sediments in the Usumacinta River’s middle (left) and lower (right) basins during the dry (top) and rainy (bottom) seasons.

The EF values for Cd, Cr, Ni, and V were >10 in several cases. In contrast, Se was consistently >10 across all stations, seasons, and the middle and lower basins, suggesting that sediments are enriched in Se. Specifically, during the dry season, Se and Cd were contaminated or enriched (EF ≥ 10) at all sites, whereas Sb was enriched at 14 sites, As at 9, and Ni at 2. During the rainy season, Se remained enriched (EF ≥ 10) at all sites, whereas As was enriched at two sites. These results align with those previously observed in the outlier identification exercise, confirming that Se, As, Sb, and Cd are among the most enriched elements due to natural or/and human activities.

During the dry season, EF values for Se were relatively low (58 ± 34 in the middle basin and 80 ± 34 in the lower basin) compared with the rainy season (129 ± 38 in the middle basin and 130 ± 44 in the lower basin), likely indicating the presence of Se-enriched sediments. Cd showed a similar pattern, with EF values higher during the dry season (15.2 ± 3.3 in the middle basin and 23.5 ± 5.5 in the lower basin) than in the rainy season (3.8 ± 1.8 in the middle basin and 4.0 ± 1.4 in the lower basin). This contrast between dry and rainy periods may be due to the contribution of particulate matter released and carried away during rainfall, which contains these elements. It is well known that forest fires and biogenic (vegetation) sources release particles containing heavy metals (Nriagu, 1990) into the atmosphere as windblown dust; these particles are subsequently transported and deposited. At least part of the Usumacinta basin lies downwind from the Central American Volcanic Arc; therefore, the ash could add to the metal(loid) load. The chemical makeup of ash varies significantly between ecosystems and even within them; some ash shows high levels of metals like Mn, Zn, and Pb (Sánchez-García et al., 2023).

A key aspect of defining metal(loid) anomalies is establishing the geochemical background, given the complexity and diversity of processes that influence sediment composition (Bábek et al., 2015). The approach, known as the “global average values”, uses global-average upper-crust and marine-shale metal concentrations as standard reference materials, which are widely used. However, their limitation is that they do not necessarily reflect local conditions (Birch, 2017), as might be the case in this study with Se, potentially leading them to overlook the heterogeneity of the metal(loid) basin composition. Several methods are available to determine metal(loid) background levels and enrichment in sediments, classified as empirical and statistical methods.

Among the six empirical methods reviewed (global values, pristine marine and fluvial sediments, catchment soils, and rocks) by Birch (2017), it is concluded that using sediment cores offers the most significant advantage because it not only provides background levels but also creates a contamination record and determines the start of pollution. In our study area, information from a ^210^Pb-dated core (Ruiz-Fernández et al., 2019) sampled near the mouth of the Usumacinta River was examined to evaluate EFs in an alternative manner. The background values reported for this core (As 6.3, Ba 286.6, Cr 152.2, Cu 24.1, Ni 74.6, Pb 13.2, V 64.6, and Zn 60.4 µg g^−1^) were compared with those reported in the current study. These values were equal to or higher than the concentrations found in the Usumacinta River, which confirms that the strategy using values from the Earth’s crust of the surficial rocks (Martin & Meybeck, 1979) and the average shale concentration (Taylor, 1964) may be applicable in the study area at least for As, Ba, Cr, Cu, Ni, Pb, V, and Zn. The main issue with using the background data is that this core was collected from a location just off the mouth of the Usumacinta River, on the continental shelf, in an area affected by the petroleum industry. Another factor of consideration is the role of Mn, which, during diagenesis under reduced conditions, could significantly affect the levels of meta(loid)s in the sedimentary column (Birch, 2017).

***Assessment by the Igeo index.*** The metal(loid) Igeo values from this study varied widely (Fig. 4). The Igeo classification suggests that stations with Igeo <0 are uncontaminated, as seen for Ag, Ba, Be, Cd, Mn, Sb, and Tl. Those (As, Co, Cr, Cu, Ni, Pb, Sn, V, and Zn) with 0 <Igeo <1 are uncontaminated to moderately contaminated. In contrast, during the rainy season, Se reached Igeo values greater than 5 at twelve sites (M1, M3, M4, M5, M7, M8, L1, L2, L4, L5, L7, and L9), suggesting highly contaminated or enriched conditions (Singh et al., 2017). These Igeo results (coinciding with EFs) suggest no significant contamination by most metal(loid)s examined, except for Se at 14 analyzed sites.

**Figure 4:**
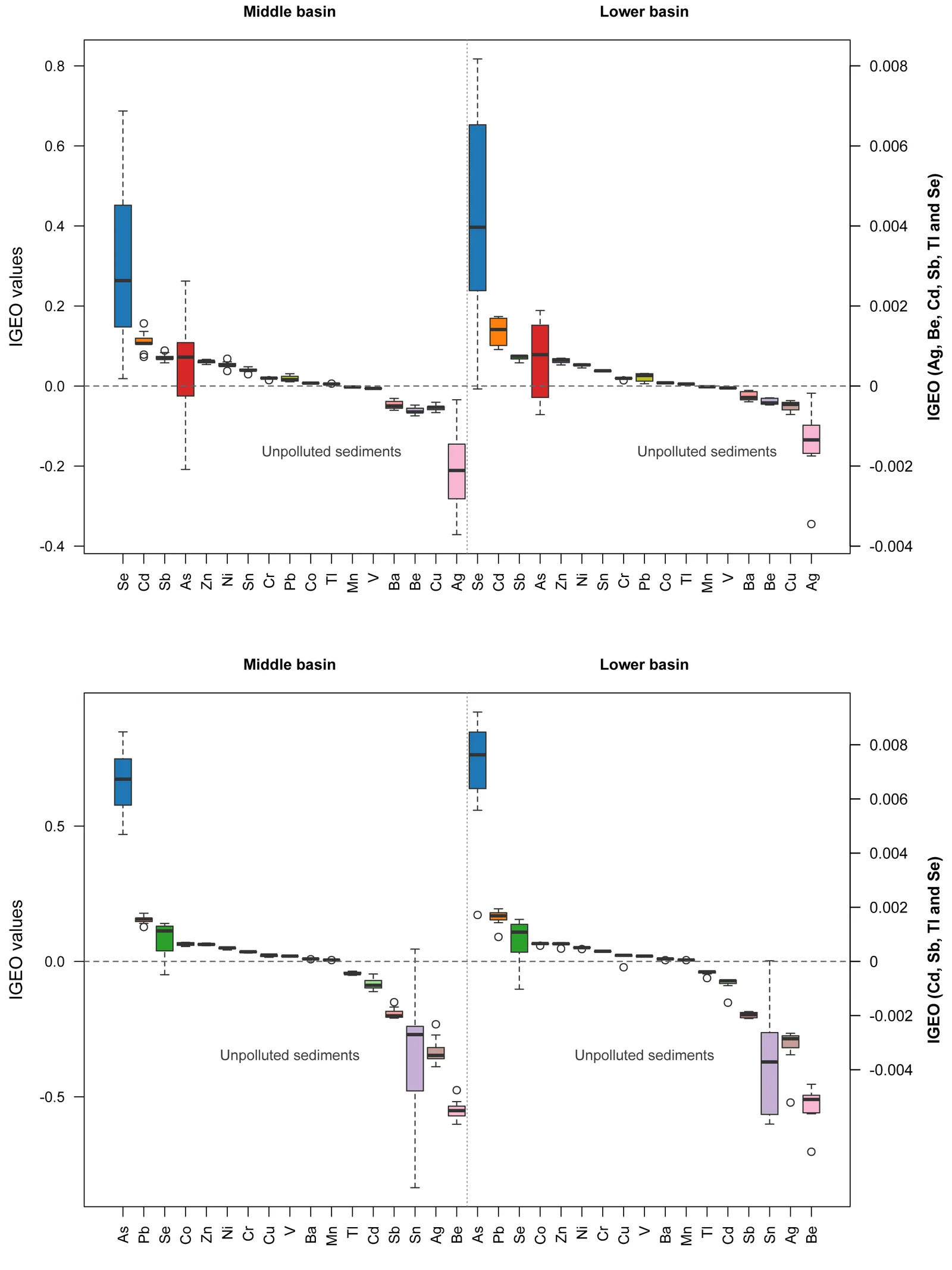
Geo-accumulation index (Igeo) for sediments in the Usumacinta River’s middle (left) and lower (right) basins during the dry (top) and rainy (bottom) seasons.

The EF and Igeos results for Se suggest that some natural process in the middle and lower basins of the Usumacinta River enriches this metalloid. Se contents in the middle and lower basin sites during the two seasons are relatively elevated compared to the Se content in the Earth’s crust, which has an abundance of 0.13 µg g^−1^ (Rudnick & Gao, 2003) or a 0.29 µg g^−1^ background (Buchman, 2008). These relatively high concentrations could be linked to Se in the parent rocks, which serve as a rich source of this element. The element is released through weathering and erosion and then transported downstream by water. This Se-rich pattern has been observed in other parts of the world, where the presence of Se-rich soils has been connected to the parent material of the Yuanzhou district of the Loess Plateau, China (Li et al., 2023), and in the Lujiaping Formation of lower Cambrian strata from Naore Valley, China (Pinzón-Nuñez et al., 2023).

The co-enrichment of Se and Cd in the sediments of the Usumacinta River, especially during the dry season at all sites, can be attributed to the parent rocks in the upper basin. These rocks include Quaternary alluvium, gypsum, marl areas, and regions with Cretaceous carbonates and marine sediments. The middle basin consists of marine and continental clastic-carbonate sequences, Cenozoic volcanic deposits (Morán-Zenteno et al., 1999), dolomites, and Cretaceous limestones (Marshall, 2007). Carbonates and marine sediments are often associated with Se enrichment and, in some cases, with co-enrichment of Cd, as observed in Southern China, where moderate to high levels of these two elements are reported (Han et al., 2024).

The occurrence of Se enrichment has been explained mainly in three ways (Tabelin et al., 2018): (i) Se concentrated in sulfide minerals associated with uranium ores in sandstone-type deposits and in similar metal deposits in sedimentary rocks; (ii) seleniferous (Se-rich) coals and other forms of fossil fuels such as crude oil and oil shale; and (iii) Se content in volcanic rocks.

There are three mechanisms through which sedimentary rocks gain Se: (1) weathering of continental rocks, (2) sequestration from seawater, and (3) formation of evaporite salts during sedimentation (Tabelin et al., 2018). In the Usumacinta basin, there is evidence of dolomites and ancient marine deposits (Morán-Zenteno et al., 1999; Marshall, 2007) that could favor the concentration of Se under the proposed mechanism. An alternative explanation for the Se-enrichment is the hydrothermal origin (Long & Luo, 2017), as the correlations between Se and Ba were significant (*p* < 0.05) in the study area during both the dry and rainy seasons (Supplementary Information (Fig. S4)). Figure 5 illustrates a conceptual model summarizing the main factors controlling differences in the enrichments of selenium and cadmium in the middle and lower Usumacinta River basin.

**Figure 5:**
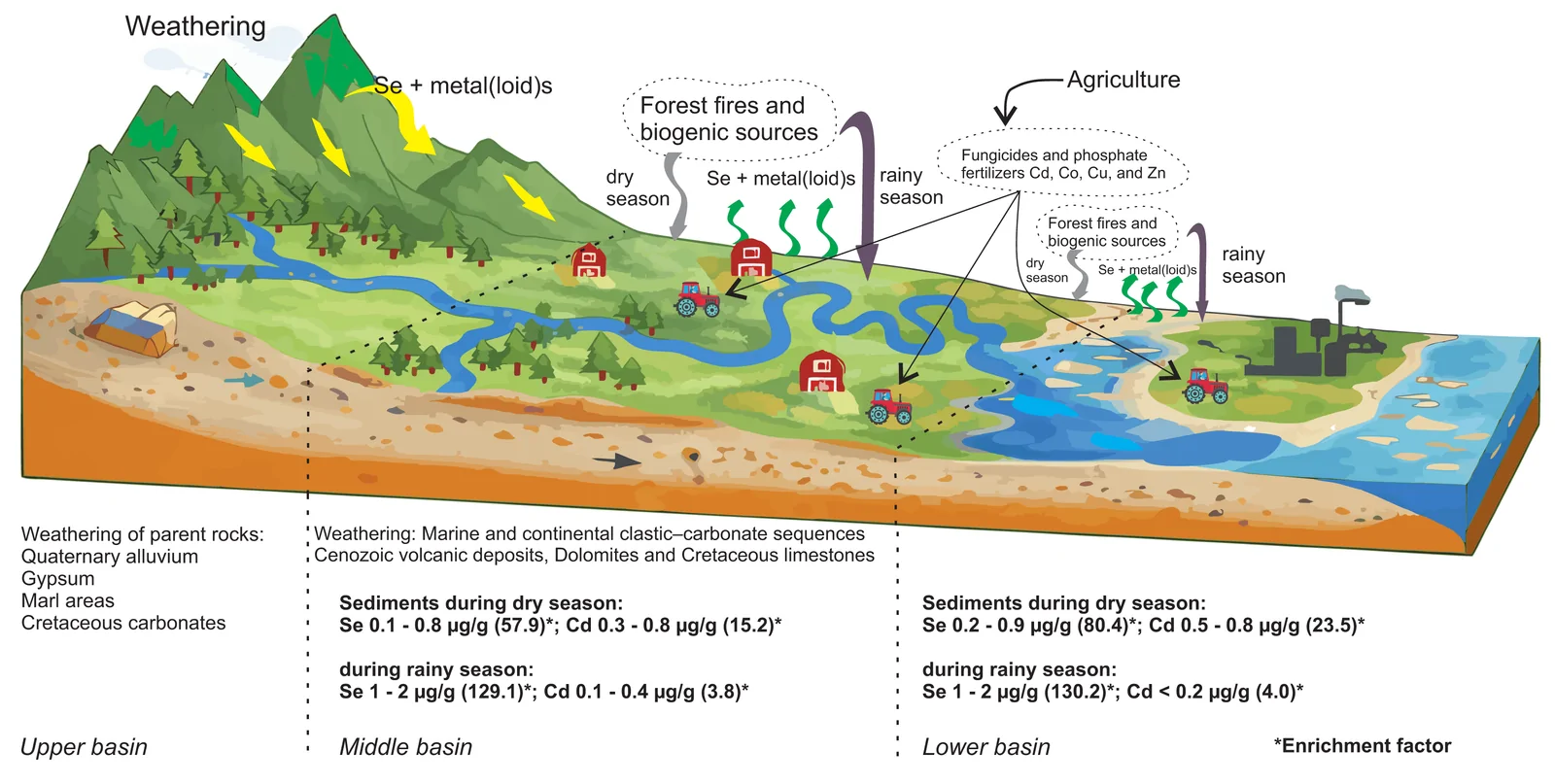
Conceptual model that control the enrichment of selenium in Usumacinta River. Conceptual model summarizing the main factors that control the differences between the enrichments of selenium and metal(loid)s in the middle and lower Usumacinta River basin.

***Assessment by CF, CD, and PLI index.*** The metal(loid) CF levels in this study were relatively low (Fig. 6). In contrast, CD consistently fell into classes 2, 3, and 4 in both basins during the rainy season, with a tendency to increase to class 4 during this period (Fig. 7). Results show that 12 of 17 sites during the dry season and all 17 sites during the rainy season are contaminated with Se. The PLI is less commonly used but can be applied to a sampling area that integrates all analyzed metal(loid)s. During the dry season, the PLI values in this study ranged from 0.25 to 0.48 in the middle basin and from 0.21 to 0.53 in the lower basin. During the rainy season, PLI values were similar in the middle (0.25–0.34) and lower (0.14–0.41) basins (Supplementary Information (Table S5)). Slightly higher PLI values are observed in the lower basin compared to the middle basin during both seasons (Fig. 7), suggesting that the former is somewhat further from a non-polluted condition than the latter. However, these values suggest that both basins of the Usumacinta River are in an intermediate state between a completely unpolluted condition (PLI = 0) and the pollutant baseline levels (PLI = 1), with no station showing contamination in either the dry or rainy seasons.

**Figure 6:**
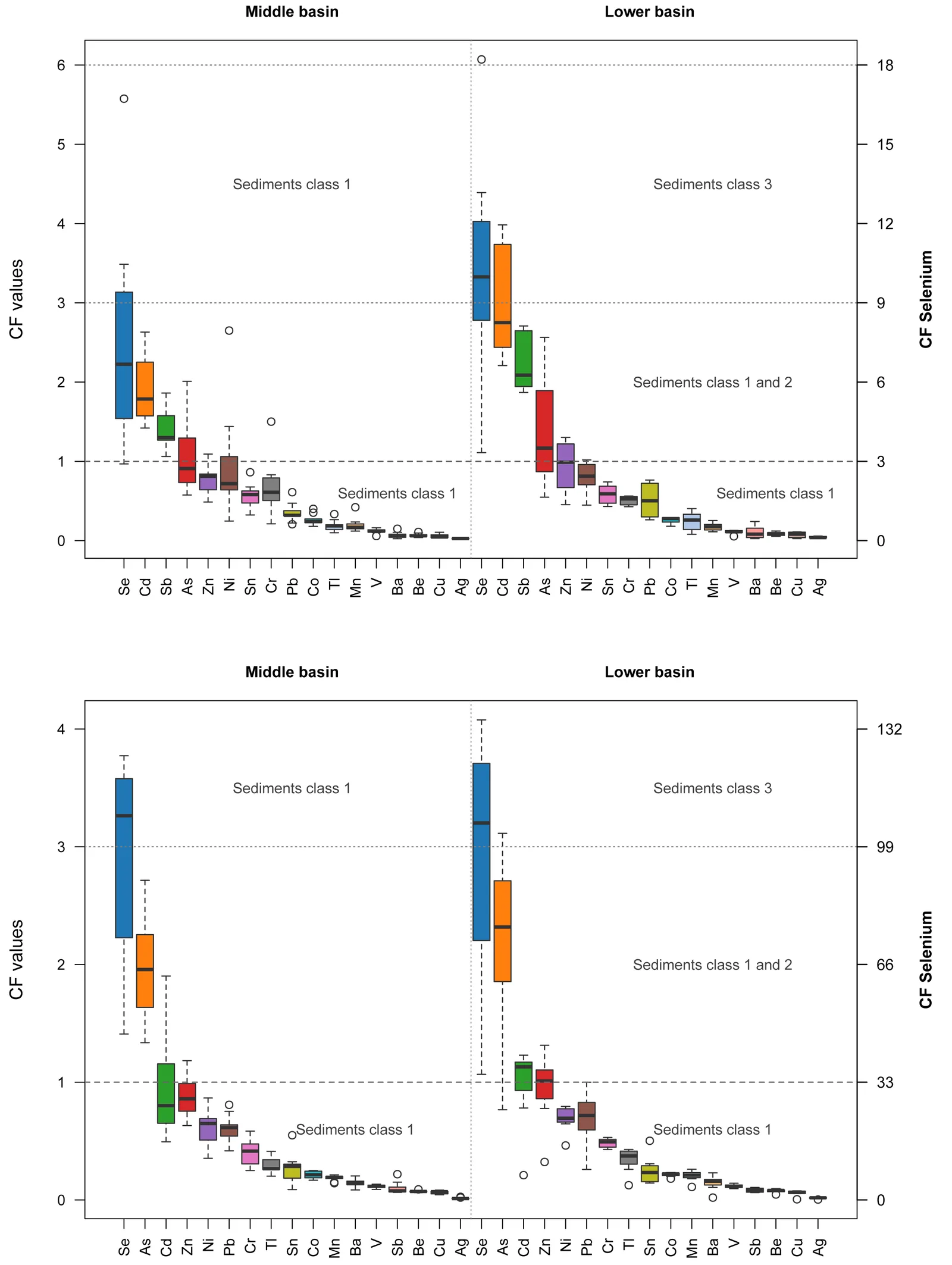
Contamination factor (CF) for sediments in the middle (left) and lower (right) basins of the Usumacinta River during the dry (top) and rainy (bottom) seasons.

**Figure 7:**
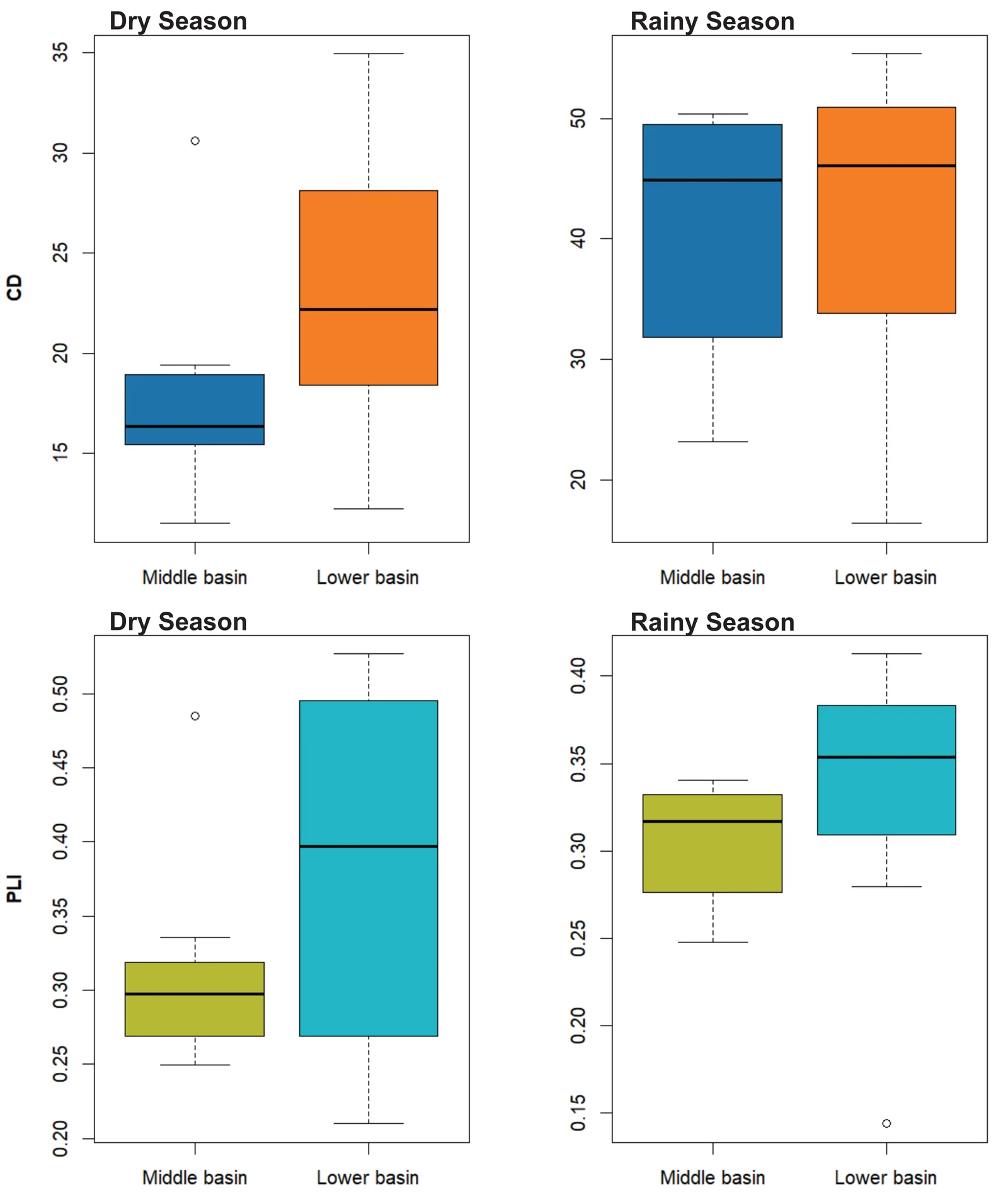
Contamination degree (CD) (above) and pollution load index (PLI) (below) for sediments in the Usumacinta River’s middle and lower basins during the dry (left) and rainy (right) seasons.

### Metal(loid) sources

The geology of the upper basin of the Usumacinta River features extensive Quaternary alluvium, gypsum, and marl deposits, as well as Cretaceous and marine carbonate sediments (Morán-Zenteno et al., 1999). In contrast, the middle basin is a mountainous region composed of Cretaceous limestones, dolomites, and marine and continental carbonates, as well as Cenozoic volcanic deposits. The Usumacinta River drains the Sierra de Los Cuchumates in northern Guatemala through the folded Cretaceous limestone of the Sierra de San Cristobal, where its drainage pattern interlaces with numerous geomorphic features, such as sinkholes caused by limestone dissolution (Hudson et al., 2005). The coastal plains of the lower basin consist of lowlands composed of Quaternary alluvial, coastal, lacustrine, and palustrine sediments, along with Pleistocene sedimentary materials such as marls, sandstone-shales, and carbonate rocks. These sediments result from the denudation of the hilly region (Hudson et al., 2005).

The Usumacinta River basin contains various lithological units that affect the distribution and leachability of metal(loid)s in surface sediments. Significant geological formations include granite and diorite, which are key sources of several metal(loid)s due to their mineralogy and weathering processes. Granite, a common rock type in the basin, contains metalloid elements such as As, Cd, and Cu. Weathering of granitic rocks can release these elements into the environment, with As and Cd being especially mobile in acidic conditions (Alloway, 2013). Copper is also frequently found in granites (Nagajyoti, Lee & Sreekanth, 2010) and can be released into river systems through physical and chemical weathering processes, especially in areas with high rainfall or fluctuating pH levels.

Diorite, another common rock type in the Usumacinta basin, contains Cr, a metal typically associated with acidic igneous rocks such as diorite and granite (Alloway, 2013). The leachability of Cr in the environment depends strongly on pH and redox conditions, with more mobile forms of Cr released under acidic conditions. Additionally, diorite and granite are characterized by high Si content, and higher Si content in silicate rocks is associated with increased Pb concentrations (Alloway, 2013). Antimony in the basin may also be significant, as this element is commonly associated with sulfide minerals such as stibnite, antimonite, and valentinite, which are typically found in volcanic rocks and are linked to Cu, Ag, and Pb ores (Butterman & Carlin, 2004).

Regarding leachability, climate, pH, and weathering intensity, these factors are key in determining the migration of metal(loid)s from rocks to surface sediments. For example, regions with higher rainfall or fluctuating pH levels will undergo more intense weathering, which increases the release of metal(loid)s into the river system. Conversely, agricultural activities and land-use changes can significantly introduce metals and nutrients into rivers. Important processes include the use of fertilizers and pesticides in farming, which often contain As, Cd, Cu, Pb, and Zn, and deforestation that leads to soil erosion and sediment transport, potentially carrying both natural and human-mobilized metals (Páez-Osuna et al., 2017; Srivastava et al., 2017; Caili et al., 2020).

The results suggest that in the Usumacinta River basin, the principal source of metal(loid)s is from weathered crust material. However, forest fires and biogenic sources could also contribute significantly, as they do elsewhere (Nriagu, 1990); particles released by erosion or vegetation appear in the atmosphere as windblown dust. Then, it is probable that a fraction of the deposited metal(loid)s in the sediments of the middle and lower basin of the Usumacinta River is also of this last origin.

In summary, the persistence of elevated Se concentrations during both seasons suggests a dominant geogenic background linked to the weathering of Cretaceous marine sedimentary rocks, as observed in other regions (Presser & Swain, 1990; Fordyce, 2013). However, the stronger co-enrichment of Se and Cd observed during the dry season, when river discharge and dilution are minimal, is consistent with an additional anthropogenic contribution, potentially related to phosphate-based agricultural inputs. Rather than being mutually exclusive, these patterns likely indicate a superposition of a regional geogenic baseline and a seasonally modulated anthropogenic signal.

The dry season PCA (Fig. S5) explained 77% of the cumulative variance in the first two principal components (Table S0d) with PC1 accounting for 60.7% and PC2 for 16.3%. During the dry season, Al, Ba, Pb, Tl, and Zn had the highest scores on the positive side of PC1, whereas Sn had the highest score on the negative side. Co and Cr had the highest scores on the positive side of PC2, while Sb had the highest on the negative side. The rainy season PCA (Fig. S5) accounted for 76.8% of the cumulative variance in the first two principal components, with PC1 accounting for 58.3% and PC2 for 18.5%. In this season, Al, Pb, Tl, and Zn had the highest scores on the positive side of PC1, whereas Sn had the highest score on the negative side. Co and Cr had the highest scores on the positive side of PC2, while Sb and Sn were on the negative side. The distribution of factor scores in the two biplots showed partial segregation between the basin’s middle and lower regions, which was more pronounced during the rainy season (Fig. S5).

Both cluster analyses (Fig. S5) revealed a high similarity between sampling stations across both seasons. There was no clear separation between the middle and lower basins; the clusters included sampling stations from both basins. Both dendrograms displayed two clusters with a low cut level (<15). The dry season dendrogram (Fig. S5) at a lower cutting level (10) grouped a similar number of stations in each cluster (seven and ten). In contrast, the clusters were uneven during the rainy season, with three and 14 sampling stations in the dendrogram (Fig. S5) at a higher cutting level (15). However, at a lower cutting level (10), the cluster with 14 stations splits into two sub-clusters, one with six stations and the other with eight stations. The PCAs and the dendrograms agree in grouping the same stations each season. However, the sampled stations in each cluster differed between seasons. The most important metals (*i.e.,* those with higher loadings; Table S0d) explaining the common grouping in both seasons are Al, Co, Cr, Pb, Sb, Sn, Tl, and Zn, suggesting that these metal(loid)s are mainly associated with silicate-aluminates.

### Environmental implications

The relatively high Se concentrations found in the Usumacinta River basin, linked to Se in the parent rocks, have been observed in other parts of the world (*e.g.*, China, Li et al., 2023; Pinzón-Nuñez et al., 2023; USA, Presser & Luoma, 2004; Rosen et al., 2023), and can have environmental implications of variable magnitude. A particularly significant example is that experienced by the southwestern region of the United States. Quaternary marine and continental shales in the western United States are sources of Se that have been loaded into the aquatic environment through mining, agricultural, and energy production (Rosen et al., 2023). The mobilization of Se from shales through agricultural irrigation has been recognized since the 1930s; however, the discovery of deformities in birds and other wildlife during 1980s spurred studies to determine the extent and effects of the Se contamination. Studies found that the biota of the Salton Sea drainage basin were at risk from legacy Se contamination in the Colorado River watershed (Rosen et al., 2023). Presser & Luoma (2004) detail that marine sedimentary rocks of the Coast Ranges contribute Se to soil, surface water, and groundwater in the western San Joaquin Valley, California. To understand the effects of Se inputs, they developed the Bay-Delta Selenium Model. However, the human health implications of Se in California’s San Joaquin Valley have been studied since the 1980s; Fan et al. (1987) evaluated the effects of elevated Se levels in agricultural wastewater, fish, and waterfowl. Also found that soil Se concentrations are highly variable, suggesting a potential source of high exposure.

The Se-Cd co-enrichment needs to be identified and defined in the middle and lower basins of the Usumacinta River, as this may also result in the co-enrichment of Se and Cd in soils undergoing intense chemical weathering (Wang et al., 2019). Remarkably, the co-enrichment of Se and Cd in soils may lead to agro-foods becoming contaminated with both Cd and Se (Han et al., 2024), potentially posing health risks to consumers. Several recommendations have been proposed for managing Se-rich land (Han et al., 2024), including restricting the use of contaminated Se-rich land for agriculture, adopting a cautious approach to using lightly polluted Se-rich land, and promoting the development of Se-rich agriculture in uncontaminated Se-rich areas. Moving forward, developing technologies that enhance Se and Cd absorption is crucial for the safe utilization of Se-rich lands affected by Cd pollution.

## Conclusions

In general, results showed that metal(loid) concentrations were higher during the dry season than during the rainy season, and that concentrations in the lower basin were higher than in the middle basin. No significant spatial or seasonal differences were observed in the concentrations of Be, Cu, Fe, Li, Mg, Mn, Ni, and Sr. Conversely, significant spatial and temporal differences were found in Ag, Al, As, Ba, Cd, Cr, Co, Pb, Sb, Se, Tl, Sn, V, and Zn. According to the multivariate analyses (PCA and cluster), there was no clear separation between the middle and lower basins in either season. Except for Se, the metal(loid)s found in the Usumacinta River sediments are similar to those reported from other tropical rivers, but with lower concentrations or within the lower reported range.

The high Se levels (up to 2.0 µg g^−1^) are consistent with the weathering of Cretaceous black shales and seleniferous limestones present in the upper basin (Guatemala/Chiapas). Although the agriculture practiced in most parts of the Usumacinta basin is underdeveloped, it covers an extensive area and could also contribute significantly to Cd and, eventually, to Se through agricultural runoff.

Generally, the EFs of most elements and stations were less than 1.5, suggesting that such metal(loid)s are derived from natural weathering. Conversely, the EFs of As, Co, Mn, Pb, Sb, Sn, and Zn across sites ranged from 1.0 to 10, likely indicating moderately contaminated sediments. The EFs of Se exceeded 10 at all stations, across all seasons, and in both basins, suggesting that sediments are enriched by natural processes associated with the parent rock. The Igeo values supported this, as Se showed Igeo values exceeding 5 at 12 sites during the rainy season, suggesting anomalous conditions. These results partially confirmed the first and second hypotheses regarding metal(loid) variability across the basins and seasons. Finally, As, Co, Cr, Cu, Ni, Pb, Sn, V, and Zn showed Igeo values suggesting sediments that are uncontaminated to moderately contaminated, partially confirming the third hypothesis. Future studies require more detail on the extreme lower basin of the Usumacinta River, where anthropogenic activities have been moderate to intensive.

##  Supplemental Information

10.7717/peerj.21776/supp-1 Supplemental Information 1 Data and supplemental figures and tables

## Additional Information and Declarations

### Data Availability

The following information was supplied regarding data availability: The raw measurements are available in the Supplemental Files.
