# Supplementary material for: Metal(loid)s in surface sediments of the middle and lower basins of the Usumacinta River (southeastern Mexico): variability and selenium enrichment
Source: PeerJ. 2026 Oct 5;14:e21776. doi: 10.7717/peerj.21776 (PMC13648217; doi:10.7717/peerj.21776)
Supplement: Supplementary file 1 [file peerj-14-21776-s001.docx]

**Supplementary information (SI)**

Article title: **Metal(loid)s in surface sediments of the middle and lower basins of the Usumacinta River (southeastern Mexico): variability and selenium enrichment**

Journal name: **PeerJ**

Author names:

Javier Alcocer^1^, Federico Páez-Osuna^2,^*, Selene Olea-Olea^3^, Luis A. Oseguera^1^, Raul Silva-Aguilera^3^

^1^ Grupo de Investigación en Limnología Tropical, FES Iztacala, Universidad Nacional Autónoma de México, Tlalnepantla, Estado de México, Mexico**.**

^2^ Unidad Académica Mazatlán, Instituto de Ciencias del Mar y Limnología, Universidad Nacional Autónoma de México, Mazatlán, Sinaloa, Mexico.

^3^ Departamento de Dinámica Terrestre Superficial, Instituto de Geología, Universidad Nacional Autónoma de México, Ciudad de México, Mexico.

Email address of the corresponding author: [paezos@ola.icmyl.unam.mx](mailto:paezos@ola.icmyl.unam.mx)

**Procedure for the selection of normalizer**

The selection of normalizer was made based on the criteria described by Soto-Jimenez and Páez-Osuna (2001), considering the best correlation between each potential normalizer (Al, Fe, and Li) and the granulometry and organic matter; clearly, Al showed the best correlations in both the lower and middle basins (Figs. S1). In brief, this consists of evaluating the correlations between normalizing elements and metals examined using regression analysis with all the data points. Points outside the 95% prediction limits were removed to avoid the inclusion of any sample influenced by anthropogenic metal sources in the final regression model and to determine the statistical relationship solely based on natural concentrations. In addition, the metal concentration is considered toxic to biota obtained from a biological effects database referred to as effects range-low (ERL) according to guidelines (Cd 1.2, Cr 81, Cu 34, Ni 20.9, Pb 46.7, and Zn 150 µg/g; in our case, there were no samples above or equal to such levels, were removed. Regression models between each metal to al were repeated after all statistical outliers and values exceeding ERL guidelines were eliminated, considering a “natural” relationship that includes data points from sediments with a null or very low probability of anthropogenic enrichment. The selected normalizer was made from those correlations that produced a higher regression coefficient. Furthermore, a predicted value and standard error were calculated for each metal(loid) in every sample by the regressions’ equations; these values were used to obtain a regional reference range for all points (both basins and seasons) on the regression line for each metal, which is defined as the predicted value plus twice the standard error of the estimate. The points outside the upper limit were discernible without the graphics; anthropogenic sources influenced these samples (Fig. S2).

The values for the earth's crust used were from Martin and Meybeck (1979) and represent the average composition of the surficial rocks exposed to weathering: Al 6.9%, Fe 3.6%, Cd, 0.2, Co 13, Cr 71, Cu 32, Li 42, Mn 720, Ni 49, Pb 16, V 97, and Zn 127 µg/g.

Enrichment factor criteria

An EF around 1.0 indicates that the metal(loid) in the sediment originated predominantly from lithogenous material, whereas EF>1.0 indicates that the element is of anthropogenic origin; furthermore, EF < 1.0 indicates clean sediments, 1.0 < EF ≤ 10, indicates that sediments are small or moderately contaminated, and EF > 10 are contaminated sediments. The normalizer was selected based on the criteria described by Soto-Jimenez and Páez-Osuna (2001) considering the best correlation between each potential normalizer and the granulometry, as well as the organic matter.

Geo-accumulation index criteria

The Igeo factor is composed of seven classes (0–6), indicating different metal enrichment degrees above the average shale value, ranging from unpolluted to very highly polluted sediment: Uncontaminated Igeo < 0; uncontaminated to moderately contaminated 0 < Igeo < 1; moderately contaminated sediment 1 < Igeo < 2; moderately to strongly contaminated 2 < Igeo < 3; strongly contaminated 3 < Igeo < 4; strongly to extremely contaminated 4 < Igeo < 5; and extremely contaminated Igeo >5.

Contamination factor and contamination degree criteria

The CF was calculated using the equation CF = (mean metal(loid) concentration at the contaminated site)/(metal(loid) average shale concentration), which Håkanson (1980) classified into four classes: class 1, CF < 1; class 2, 1 ≤ CF < 3; class 3, 3 ≤ CF < 6; and class 4, CF ≥ 6. Contamination degree (CD) is conventionally defined for river sediments as the sum of all CF values from a particular sampling site. Ahdy and Khaled (2009) classified the CD into sediment classes: class 1, CD < 6; class 2, 6 ≤ CD < 12; class 3, 12 ≤ CD < 24; and class 4, CD ≥ 24.

References

Soto-Jimenez. M.F., Páez-Osuna F. 2001. Distribution and normalization of heavy metal concentrations in mangrove and lagoonal sediments from Mazatlan Harbor (SE Gulf of California). Estuarine, Coastal and Shelf Science 53, 259-274. https://doi.org/10.1006/ecss.2000.0814

**References on the Servicio Geológico Mexicano SGM: SGM 1996; 1997a; 1997b; 1997c; 1997d; 1997e; 1998**

SGM (Servicio Geológico Mexicano), 1996. Carta Geológico-Minera. Villa Hermosa. E15-8. https://mapserver.sgm.gob.mx/Cartas_Online/geologia/106_E15-8_GM.pdf

SGM (Servicio Geológico Mexicano), 1997a. Carta Geológico-Minera. Tenosique. E15-9. https://mapserver.sgm.gob.mx/Cartas_Online/geologia/113_E15-9_GM.pdf

SGM (Servicio Geológico Mexicano), 1997b. Carta Geológico-Minera. Tuxtla Gutierrez. E15-11. https://mapserver.sgm.gob.mx/Cartas_Online/geologia/107_E15-11_GM.pdf

SGM (Servicio Geológico Mexicano), 1997c. Carta Geológico-Minera. Huixtla. D15-2. https://mapserver.sgm.gob.mx/Cartas_Online/geologia/108_D15-2_GM.pdf

SGM (Servicio Geológico Mexicano), 1997d. Carta Geológico-Minera. Frontera. E15-5. https://mapserver.sgm.gob.mx/Cartas_Online/geologia/105_E15-5_GM.pdf

SGM (Servicio Geológico Mexicano), 1997e. Carta Geológico-Minera. Las Margaritas. E15-12 D15-3. https://mapserver.sgm.gob.mx/Cartas_Online/geologia/114_E15-12-D15-3_GM.pdf

SGM (Servicio Geológico Mexicano), 1998. Carta Geológico-Minera. Ciudad del Carmen. E15-6. https://mapserver.sgm.gob.mx/Cartas_Online/geologia/112_E15-6_GM.pdf

**Fig. S1**. Grain size distribution (*above*) and organic matter (SOM) (*below*) content in the sediments of the Usumacinta River during the rainy and dry seasons (the red dotted line indicate the average value).

**Fig. S2**. Metal(loid)s spatial distribution in Usumacinta River sediments in the middle (M) and lower (L) basins during the dry (yellow bars) and rainy season (blue bars).

**Fig. S3**. Grain size (mz) to aluminum correlation in the Usumacinta River sediments during the dry (A) and rainy (B) seasons and organic matter (SOM) to aluminum correlation in the Usumacinta River sediments during the dry (C) and rainy (D) seasons. (Grey squares: middle basin, black circles: lower basin).

**Fig. S4**. Barium to selenium correlation in the Usumacinta River sediments during the dry (top) and rainy (bottom) seasons.

**Fig. S5**. Principal component analysis (PCA) of metal(loid) concentrations in surface sediments along the Usumacinta River during the dry (A) and rainy (B) seasons, along with cluster analysis of metal(loid) concentrations in sediments during the dry (C) and rainy (D) seasons.

**Tables**

**Table S0a**. Quality data control of elements concentrations determined in the Usumacinta River by using the reference material IAEA-158 marine sediment: mean, standard deviation (SD), coefficient of variation (CV), recovery (%), and detection limit (DL).

| Element | Mean (µg g^-1^) | SD | CV (%) | Recommended concentration of the IAEA-158 (µg g^-1^) | Recovery (%) | DL  (µg L^-1^) |
| --- | --- | --- | --- | --- | --- | --- |
| Ag | 0.199 | 0.02 | 10.0 | 0.180 | 110.6 | 0.008 |
| Al** | 52.5 | 4.5 | 8.6 | 51.8** | 101.4 | 0.255 |
| As | 10.5 | 0.4 | 3.8 | 11.5 | 91.3 | 0.132 |
| Ba* | 1115 | 67 | 6.0 | 1028 | 108.5 | 0.265 |
| Cd | 0.360 | 0.012 | 3.3 | 0.372 | 96.8 | 0.003 |
| Co | 9.0 | 0.5 | 5.6 | 9.2 | 97.8 | 0.045 |
| Cr | 71.9 | 5.9 | 8.2 | 74.4 | 96.6 | 0.096 |
| Cu | 51.1 | 1.9 | 3.7 | 48.3 | 105.8 | 0.035 |
| Fe** | 28.3 | 2.0 | 7.1 | 26.3** | 107.6 | 0.027 |
| Li* | 30.0 | 1.9 | 6.3 | 32.5 | 92.3 | 0.205 |
| Mg* | 12195 | 1050 | 8.6 | 10390 | 117.4 | 0.266 |
| Mn | 381 | 22 | 5.8 | 356 | 107.0 | 0.019 |
| Ni | 26.9 | 1.1 | 4.1 | 30.3 | 88.8 | 0.066 |
| Pb | 36.5 | 1.4 | 3.8 | 39.6 | 92.2 | 0.002 |
| Sb | 1.02 | 0.11 | 10.8 | 1.34 | 76.1 | 0.055 |
| Sn | 5.14 | 0.52 | 10.1 | 5.84 | 88.0 | 0.095 |
| Sr | 551 | 32 | 5.8 | 473 | 116.5 | 0.305 |
| V | 66.4 | 3.6 | 5.4 | 73.0 | 91.0 | 0.066 |
| Zn | 163.1 | 8.2 | 5.0 | 140.6 | 116.0 | 0.078 |

*Classification assigned to the value for analyte as information value; ** in mg g^-1^.

**Table S0b**. Quality data control of elements concentrations determined in the Usumacinta River by using the reference material IAEA-433 marine sediment: mean, standard deviation (SD), coefficient of variation (CV), and recovery (%).

| Element | Mean (µg g^-1^) | SD | CV (%) | Recommended concentration of the IAEA-433 (µg g^-1^) | Recovery (%) |
| --- | --- | --- | --- | --- | --- |
| Ag | 0.148 | 0.015 | 10.1 | 0.133 (0.127-0.139) | 111.3 |
| Al** | 77.9 | 5.9 | 7.6 | 78.2 (76.8-79.6)** | 99.6 |
| As | 20.3 | 1.1 | 5.4 | 18.9 (18.4-19.4) | 107.4 |
| Ba | 299 | 30 | 10.0 | 268 (241-295) | 111.6 |
| Cd | 0.140 | 0.01 | 7.1 | 0.153 (0.145-0.161) | 91.5 |
| Co | 12.5 | 0.9 | 7.2 | 12.9 (12.6-13.2) | 96.9 |
| Cr | 133 | 5.9 | 4.4 | 136 (134-138) | 97.8 |
| Cu | 29.5 | 1.4 | 4.7 | 30.8 (30.2-31.4) | 95.8 |
| Fe ** | 47.5 | 2.1 | 4.4 | 40.8 (40.3-41.3)** | 116.4 |
| Li | 61.1 | 5.9 | 9.7 | 67.0 (65.4-68.6) | 91.2 |
| Mg** | 13.9 | 1.6 | 11.5 | 11.5 (11.1-12.0)** | 120.9 |
| Mn | 337 | 19 | 5.6 | 316 (312-320) | 106.6 |
| Ni | 34.9 | 2.7 | 7.7 | 39.4 (38.7-40.1) | 88.6 |
| Pb | 23.9 | 0.9 | 3.8 | 26.0 (25.4-26.6) | 91.9 |
| Sb | 1.77 | 0.23 | 13.0 | 1.96 (1.89-2.03) | 90.3 |
| Se | 0.69 | 0.07 | 10.1 | 0.78 (0.70-0.85) | 88.5 |
| Sn | 2.10 | 0.33 | 15.7 | 2.32 (2.17-2.47) | 90.5 |
| Sr* | 343 | 29 | 8.4 | 302 (296-308) | 113.6 |
| V | 149 | 6.8 | 4.6 | 160 (156-164) | 93.1 |
| Zn | 109 | 6 | 5.5 | 101 (99-103) | 107.9 |

*Classification assigned to the value for analyte as information value; ** in mg g^-1^; between parentheses is included the 95% C.I.

**Table S0c**. Quality data control of elements concentrations determined in the Usumacinta River by using the reference material NBS-1646 estuarine sediment: mean, standard deviation (SD), coefficient of variation (CV), and recovery (%).

| Element | Mean (µg g^-1^) | SD | CV (%) | Certified concentration of the NBS-1646 (µg g^-1^) | Recovery (%) |
| --- | --- | --- | --- | --- | --- |
| Al | 57.2 | 4.3 | 7.5 | 62.5 | 91.5 |
| As | 12.7 | 0.5 | 3.9 | 11.6 | 109.5 |
| Be* | 1.3 | 0.1 | 7.7 | 1.5* | 86.7 |
| Cd | 0.41 | 0.02 | 4.9 | 0.36 | 113.9 |
| Co | 12.5 | 0.6 | 4.8 | 10.5 | 119.0 |
| Cr | 71 | 2.9 | 4.1 | 76 | 93.4 |
| Cu | 20.3 | 0.9 | 4.4 | 18 | 112.8 |
| Fe ** | 37.1 | 1.9 | 5.1 | 33.5** | 110.7 |
| Li* | 44.3 | 27 | 6.1 | 49* | 90.4 |
| Mg** | 13.3 | 1.5 | 11.3 | 10.9 ** | 122.0 |
| Mn | 396 | 23 | 5.8 | 375 | 105.6 |
| Ni | 29.3 | 2.4 | 8.2 | 32 | 91.6 |
| Pb | 30.9 | 1.1 | 3.6 | 28.2 | 109.6 |
| Sb | 0.36 | 0.02 | 5.6 | 0.4 | 90.0 |
| Se* | 0.5 | 0.05 | 10.0 | 0.6* | 83.3 |
| Tl* | 0.45 | 0.02 | 4.4 | 0.5* | 90.0 |
| V | 89.5 | 6.8 | 7.6 | 94 | 96.1 |
| Zn | 129 | 6 | 4.6 | 138 | 93.5 |

*Classification assigned as non-certified; ** in mg g^-1^.

**Table S0d.** Principal component loading of trace metal concentration in the sediments along the Usumacinta River during the dry and rainy seasons (highest values in boldface).

| Element | PC1_dry_ | PC2 _dry_ | PC1_rainy_ | PC2_rainy_ |
| --- | --- | --- | --- | --- |
| Al | **0.34** | 0.04 | **0.33** | 0.18 |
| Ag | 0.24 | -0.17 | 0.31 | -0.16 |
| As | 0.29 | 0.20 | 0.31 | -0.11 |
| Ba | **0.32** | -0.04 | 0.30 | -0.04 |
| Cd | 0.27 | 0.01 | 0.30 | -0.17 |
| Co | 0.01 | **0.56** | 0.14 | **0.53** |
| Cr | -0.07 | **0.61** | -0.05 | **0.55** |
| Pb | **0.32** | 0.02 | **0.33** | -0.07 |
| Sb | 0.15 | **-0.47** | 0.25 | **-0.31** |
| Se | 0.27 | -0.01 | 0.29 | 0.05 |
| Sn | **-0.25** | -0.07 | **-0.10** | **-0.43** |
| Tl | **0.32** | 0.08 | **0.32** | 0.11 |
| V | 0.29 | 0.13 | 0.15 | 0.09 |
| Zn | **0.33** | 0.01 | **0.33** | 0.09 |
| Standard deviation | 2.92 | 1.51 | 2.86 | 1.61 |
| Proportion of variance | 60.7% | 16.3% | 58.3% | 18.5% |

**Table S1**. Main physical and chemical characteristics (average first row, standard deviation second row) of the Usumacinta River, middle and lower basins, during the rainy and dry seasons (Cuevas-Lara et al., 2021). (Q: water discharge, Z_MAX_: maximum depth, Temp: temperature, DO: dissolved oxygen concentration, K_25_: electrical conductivity).

| Season | Basin | Q | Z_MAX_ | Temp | DO | pH | K_25_ |
| --- | --- | --- | --- | --- | --- | --- | --- |
|  |  | (m^3^ s^-1^) | (m) | (ºC) | (mg L^-1^) |  | (μScm^-1^) |
| Dry | Middle | 527 | 4.3 | 28.1 | 8.4 | 7.9 | 724 |
|  |  |  | 5.5 | 0.8 | 0.7 | 0.1 | 165 |
|  | Lower | 432 | 5.6 | 28.4 | 6.3 | 7.9 | 21,416 |
|  |  |  | 3.9 | 0.9 | 2.0 | 0.2 | 19,026 |
| Rainy | Middle | 5,934 | 6.0 | 24.5 | 5.9 | 7.7 | 356 |
|  |  |  | 5.4 | 1.3 | 2.0 | 0.2 | 84 |
|  | Lower | 2,618 | 4.8 | 27.3 | 3.0 | 7.5 | 358 |
|  |  |  | 3.7 | 0.6 | 1.5 | 0.2 | 19 |

**Table S2.** Metal(loid) concentrations (µg g^-1^) in the sediments along the Usumacinta River during the dry (up) and rainy (down) seasons and the middle (M) and lower (L) basins.

| Metal | M1 | M3 | M4 | M5 | M6 | M7 | M8 | M9 | M10 | L1 | L2 | L3 | L4 | L5 | L7 | L9 | L10 |
| --- | --- | --- | --- | --- | --- | --- | --- | --- | --- | --- | --- | --- | --- | --- | --- | --- | --- |
| Ag | 0.2 | 0.2 | 0.2 | 0.2 | 0.1 | 0.2 | 0.2 | 0.2 | 0.2 | 0.3 | 0.2 | 0.3 | 0.4 | 0.4 | 0.2 | 0.4 | 0.2 |
| Al | 10,530 | 22,590 | 5,806 | 14,640 | 4,252 | 8,726 | 10,530 | 5,165 | 6,360 | 14,300 | 3,313 | 13,550 | 23,260 | 27,340 | 8,533 | 18,250 | 4,363 |
| As | 3 | 4 | 1 | 1 | 1 | 2 | 2 | 1 | 2 | 2 | 2 | 2 | 3 | 4 | 1 | 5 | 1 |
| Ba | 31 | 64 | 10 | 20 | 34 | 25 | 44 | 18 | 17 | 32 | 15 | 38 | 65 | 68 | 18 | 103 | 11 |
| Be | 0.3 | 0.3 | 0.1 | 0.1 | 0.1 | 0.2 | 0.2 | 0.2 | 0.2 | 0.2 | 0.2 | 0.3 | 0.3 | 0.3 | 0.2 | 0.3 | 0.2 |
| Cd | 0.4 | 0.4 | 0.5 | 0.3 | 0.3 | 0.5 | 0.4 | 0.3 | 0.5 | 0.5 | 0.4 | 0.6 | 0.8 | 0.8 | 0.5 | 0.7 | 0.5 |
| Co | 5 | 9 | 6 | 10 | 6 | 7 | 6 | 5 | 6 | 7 | 5 | 7 | 7 | 7 | 7 | 7 | 5 |
| Cr | 21 | 83 | 79 | 150 | 61 | 62 | 43 | 61 | 51 | 52 | 47 | 53 | 56 | 55 | 44 | 43 | 56 |
| Cu | 6 | 6 | 2 | 2 | 2 | 2 | 4 | 3 | 3 | 4 | 2 | 5 | 6 | 6 | 3 | 6 | 1 |
| Fe | 23,550 | 37,120 | 10,230 | 45,510 | 12,500 | 23,500 | 21,660 | 19,490 | 19,300 | 25,840 | 10,460 | 25,370 | 36,840 | 40,960 | 17,990 | 35,000 | 12,190 |
| Li | 9 | 8 | 3 | 3 | 4 | 4 | 7 | 5 | 6 | 8 | 3 | 8 | 9 | 9 | 7 | 10 | 4 |
| Mg | 13,720 | 25,110 | 14,840 | 50,860 | 15,170 | 11,820 | 13,390 | 12,590 | 14,540 | 17,150 | 8,157 | 17,340 | 18,710 | 19,750 | 14,730 | 14,810 | 9,553 |
| Mn | 156 | 223 | 114 | 201 | 400 | 160 | 138 | 165 | 142 | 163 | 120 | 167 | 190 | 196 | 136 | 241 | 106 |
| Ni | 18 | 107 | 52 | 198 | 79 | 54 | 55 | 45 | 48 | 66 | 52 | 70 | 76 | 73 | 53 | 55 | 33 |
| Pb | 5 | 8 | 3 | 3 | 4 | 4 | 6 | 4 | 4 | 6 | 3 | 6 | 9 | 10 | 4 | 9 | 4 |
| Sb | 0.3 | 0.3 | 0.3 | 0.2 | 0.2 | 0.4 | 0.3 | 0.3 | 0.4 | 0.4 | 0.4 | 0.4 | 0.5 | 0.5 | 0.4 | 0.5 | 0.4 |
| Se | 0.5 | 0.8 | 0.3 | 0.3 | 0.1 | 0.2 | 0.3 | 0.5 | 0.2 | 0.5 | 0.2 | 0.4 | 0.7 | 0.9 | 0.4 | 0.5 | 0.4 |
| Sn | 1.7 | 0.8 | 1.2 | 0.9 | 1.4 | 0.6 | 1 | 1.3 | 1.2 | 0.9 | 1.3 | 1.1 | 1 | 0.9 | 1.4 | 1.5 | 1.3 |
| Sr | 22 | 127 | 74 | 92 | 34 | 55 | 47 | 39 | 55 | 180 | 22 | 78 | 103 | 118 | 44 | 76 | 30 |
| Tl | 0.12 | 0.15 | 0.08 | 0.09 | 0.05 | 0.08 | 0.09 | 0.04 | 0.06 | 0.13 | 0.04 | 0.11 | 0.17 | 0.18 | 0.08 | 0.12 | 0.05 |
| V | 17 | 18 | 14 | 15 | 8 | 16 | 16 | 22 | 20 | 15 | 7 | 15 | 17 | 17 | 13 | 17 | 14 |
| Zn | 51 | 66 | 29 | 50 | 34 | 52 | 49 | 39 | 47 | 53 | 27 | 66 | 77 | 79 | 45 | 70 | 35 |

| Metal | M1 | M3 | M4 | M5 | M6 | M7 | M8 | M9 | M10 | L1 | L2 | L3 | L4 | L5 | L7 | L9 | L10 |
| --- | --- | --- | --- | --- | --- | --- | --- | --- | --- | --- | --- | --- | --- | --- | --- | --- | --- |
| Ag | 0.07 | 0.07 | 0.1 | 0.09 | 0.08 | 0.18 | 0.14 | 0.07 | 0.06 | 0.13 | 0.12 | 0.08 | 0.12 | 0.14 | 0.13 | 0.15 | 0.02 |
| Al | 20,300 | 16,690 | 22,260 | 19,880 | 12,250 | 34,260 | 22,160 | 16,300 | 11,360 | 26,810 | 31,330 | 14,840 | 22,590 | 28,580 | 25,410 | 26,430 | 5,038 |
| As | 4 | 3 | 5 | 4 | 2 | 4 | 4 | 3 | 2 | 5 | 6 | 3 | 4 | 4 | 4 | 5 | 1 |
| Ba | 68 | 55 | 65 | 59 | 61 | 87 | 82 | 50 | 36 | 68 | 98 | 45 | 67 | 75 | 63 | 72 | 8 |
| Be | 0.3 | 0.2 | 0.2 | 0.2 | 0.2 | 0.2 | 0.2 | 0.2 | 0.2 | 0.2 | 0.3 | 0.2 | 0.2 | 0.2 | 0.2 | 0.2 | 0.1 |
| Cd | 0.1 | 0.2 | 0.2 | 0.2 | 0.1 | 0.4 | 0.2 | 0.1 | 0.1 | 0.2 | 0.2 | 0.2 | 0.2 | 0.2 | 0.2 | 0.2 | 0 |
| Co | 4 | 6 | 5 | 6 | 5 | 5 | 4 | 6 | 5 | 6 | 6 | 6 | 6 | 5 | 5 | 6 | 5 |
| Cr | 25 | 57 | 48 | 58 | 33 | 31 | 27 | 46 | 42 | 52 | 48 | 45 | 51 | 51 | 45 | 43 | 53 |
| Cu | 5 | 4 | 4 | 4 | 4 | 3 | 4 | 3 | 2 | 4 | 4 | 3 | 4 | 4 | 4 | 3 | 0 |
| Fe | 30,530 | 25,160 | 29,940 | 28,460 | 18,810 | 37,100 | 27,410 | 23,770 | 19,360 | 33,600 | 39,450 | 22,990 | 30,050 | 32,750 | 31,080 | 34,250 | 10,040 |
| Li | 9 | 7 | 8 | 7 | 7 | 7 | 7 | 6 | 6 | 8 | 7 | 6 | 7 | 8 | 7 | 8 | 4 |
| Mg | 22,230 | 24,270 | 22,420 | 23,090 | 12,320 | 10,460 | 12,670 | 15,370 | 16,060 | 21,920 | 21,340 | 17,740 | 17,290 | 18,550 | 17,760 | 16,540 | 9,990 |
| Mn | 192 | 190 | 203 | 183 | 179 | 135 | 172 | 189 | 142 | 210 | 223 | 171 | 190 | 218 | 188 | 248 | 105 |
| Ni | 26 | 62 | 51 | 65 | 48 | 37 | 38 | 52 | 46 | 59 | 50 | 52 | 58 | 57 | 51 | 48 | 35 |
| Pb | 8 | 6 | 8 | 8 | 7 | 10 | 9 | 7 | 5 | 9 | 13 | 6 | 8 | 10 | 9 | 11 | 3 |
| Sb | 0.02 | 0.01 | 0.01 | 0.01 | 0.01 | 0.03 | 0.04 | 0.02 | 0.02 | 0.02 | 0.02 | 0.01 | 0.01 | 0.01 | 0.02 | 0.02 | 0.01 |
| Se | 2 | 1 | 2 | 2 | 1 | 2 | 2 | 1 | 1 | 1 | 2 | 1 | 2 | 2 | 2 | 2 | 1 |
| Sn | 0.6 | 0.57 | 0.23 | 0.38 | 1.1 | 0.18 | 0.37 | 0.61 | 0.65 | 0.55 | 0.61 | 0.53 | 0.29 | 0.41 | 0.29 | 0.33 | 1.01 |
| Sr | 33 | 74 | 93 | 92 | 39 | 34 | 47 | 44 | 51 | 145 | 118 | 66 | 87 | 99 | 97 | 75 | 25 |
| Tl | 0.12 | 0.11 | 0.15 | 0.13 | 0.12 | 0.19 | 0.16 | 0.12 | 0.09 | 0.18 | 0.19 | 0.12 | 0.17 | 0.19 | 0.17 | 0.16 | 0.06 |
| V | 17 | 16 | 17 | 16 | 14 | 18 | 16 | 14 | 12 | 17 | 19 | 14 | 16 | 17 | 15 | 16 | 13 |
| Zn | 60 | 48 | 57 | 52 | 43 | 71 | 64 | 46 | 38 | 68 | 79 | 47 | 57 | 66 | 60 | 62 | 19 |

**Table S3**. Enrichment factors (EF) for metal(loid)s from Usumacinta River sediments in the middle (M) and lower (L) basins during the dry (up) and rainy (down) seasons.

| Metal(loid) | M1 | M3 | M4 | M5 | M6 | M7 | M8 | M9 | M10 | L1 | L2 | L3 | L4 | L5 | L7 | L9 | L10 |
| --- | --- | --- | --- | --- | --- | --- | --- | --- | --- | --- | --- | --- | --- | --- | --- | --- | --- |
| (µg g^-1^) |  |  |  |  |  |  |  |  |  |  |  |  |  |  |  |  |  |
| Ag | 0.2 | 0.11 | 0.37 | 0.13 | 0.37 | 0.30 | 0.20 | 0.34 | 0.39 | 0.22 | 0.74 | 0.23 | 0.19 | 0.17 | 0.34 | 0.23 | 0.63 |
| Al | 1 | 1 | 1 | 1 | 1 | 1 | 1 | 1 | 1 | 1 | 1 | 1 | 1 | 1 | 1 | 1 | 1 |
| As | 12.1 | 7.32 | 10.38 | 3.23 | 13.8 | 12.21 | 7.11 | 12.17 | 12.43 | 6.83 | 23.03 | 6.97 | 6.13 | 6.18 | 7.83 | 11.56 | 10.34 |
| Ba | 0.57 | 1.17 | 0.19 | 0.36 | 0.63 | 0.46 | 0.81 | 0.32 | 0.31 | 0.58 | 0.28 | 0.7 | 1.2 | 1.26 | 0.33 | 1.89 | 0.2 |
| Be | 0.85 | 0.75 | 0.4 | 0.31 | 0.42 | 0.43 | 0.58 | 0.48 | 0.52 | 0.69 | 0.42 | 0.7 | 0.77 | 0.8 | 0.57 | 0.95 | 0.46 |
| Cd | 13.96 | 16.86 | 18.19 | 11.99 | 11.1 | 20.56 | 13.96 | 12.3 | 17.6 | 21.49 | 17.26 | 21.51 | 30.62 | 31.13 | 19.11 | 27.79 | 19 |
| Co | 1.41 | 2.76 | 1.81 | 3.13 | 1.88 | 2.13 | 2.02 | 1.72 | 1.78 | 2.27 | 1.41 | 2.25 | 2.28 | 2.2 | 2.11 | 2.13 | 1.56 |
| Cr | 1.66 | 6.49 | 6.18 | 11.73 | 4.77 | 4.86 | 3.36 | 4.73 | 3.96 | 4.07 | 3.65 | 4.15 | 4.39 | 4.32 | 3.41 | 3.34 | 4.34 |
| Cu | 0.83 | 0.82 | 0.27 | 0.26 | 0.29 | 0.29 | 0.57 | 0.36 | 0.43 | 0.63 | 0.22 | 0.64 | 0.86 | 0.85 | 0.37 | 0.81 | 0.2 |
| Fe | 3.27 | 5.15 | 1.42 | 6.32 | 1.74 | 3.26 | 3.01 | 2.71 | 2.68 | 3.59 | 1.45 | 3.52 | 5.11 | 5.69 | 2.5 | 4.86 | 1.69 |
| Li | 3.43 | 3.06 | 1.17 | 1.09 | 1.63 | 1.62 | 2.73 | 1.83 | 2.37 | 3.22 | 1.36 | 3.09 | 3.59 | 3.59 | 2.72 | 4.02 | 1.55 |
| Mg | 4.6 | 8.42 | 4.98 | 17.06 | 5.09 | 3.96 | 4.49 | 4.22 | 4.88 | 5.75 | 2.74 | 5.82 | 6.28 | 6.62 | 4.94 | 4.97 | 3.2 |
| Mn | 1.29 | 1.84 | 0.94 | 1.65 | 3.29 | 1.31 | 1.14 | 1.35 | 1.17 | 1.34 | 0.99 | 1.37 | 1.56 | 1.61 | 1.12 | 1.98 | 0.87 |
| Ni | 1.92 | 11.19 | 5.39 | 20.6 | 8.23 | 5.59 | 5.72 | 4.71 | 4.97 | 6.92 | 5.41 | 7.27 | 7.91 | 7.65 | 5.55 | 5.72 | 3.48 |
| Pb | 2.96 | 4.78 | 1.74 | 1.61 | 2.47 | 2.56 | 3.69 | 2.5 | 2.44 | 3.85 | 2.05 | 4 | 5.4 | 5.97 | 2.3 | 5.92 | 2.38 |
| Sb | 12.32 | 10.07 | 12.3 | 8.61 | 8.3 | 14.33 | 9.9 | 10.15 | 14.55 | 15.47 | 14.61 | 14.92 | 20.24 | 21.17 | 15.96 | 21.14 | 16.68 |
| Se | 73.5 | 130.7 | 54.5 | 42.4 | 22.7 | 36.1 | 52.2 | 81.7 | 27.6 | 85.8 | 26 | 67.4 | 103 | 142.3 | 70.2 | 85.8 | 63 |
| Sn | 6.74 | 3.3 | 4.54 | 3.71 | 5.3 | 2.53 | 4.00 | 4.90 | 4.83 | 3.51 | 5.18 | 4.19 | 3.86 | 3.37 | 5.55 | 5.79 | 5.05 |
| Sr | 0.46 | 2.64 | 1.54 | 1.92 | 0.7 | 1.15 | 0.98 | 0.82 | 1.14 | 3.75 | 0.45 | 1.62 | 2.15 | 2.46 | 0.92 | 1.59 | 0.62 |
| Tl | 2.07 | 2.61 | 1.44 | 1.51 | 0.84 | 1.45 | 1.56 | 0.78 | 1.09 | 2.21 | 0.62 | 1.99 | 2.98 | 3.16 | 1.34 | 2.07 | 0.85 |
| V | 0.97 | 1.07 | 0.78 | 0.84 | 0.45 | 0.9 | 0.93 | 1.26 | 1.16 | 0.89 | 0.43 | 0.87 | 0.98 | 1 | 0.76 | 1 | 0.83 |
| Zn | 5.67 | 7.36 | 3.28 | 5.58 | 3.81 | 5.78 | 5.48 | 4.32 | 5.2 | 5.94 | 3.06 | 7.39 | 8.61 | 8.78 | 5.08 | 7.85 | 3.95 |

| Metal(loid) | | | | M1 | | | M3 | | | M4 | | | M5 | | | M6 | | | M7 | | | M8 | | | M9 | | M10 | | | L1 | | | L2 | | | L3 | | | L4 | | | L5 | | | L7 | | L9 | | | L10 | | |
| --- | --- | --- | --- | --- | --- | --- | --- | --- | --- | --- | --- | --- | --- | --- | --- | --- | --- | --- | --- | --- | --- | --- | --- | --- | --- | --- | --- | --- | --- | --- | --- | --- | --- | --- | --- | --- | --- | --- | --- | --- | --- | --- | --- | --- | --- | --- | --- | --- | --- | --- | --- | --- |
| (µg g^-1^) | | | |  |  |  |  |  |  |  |  |  |  |  |  |  |  |  |  |  |  |  |  |  |  |  |  |  |  |  |  |  |  |  |  |  |  |  |  |  |  |  |  |  |  |  |  |  |  |  |  |  |
| Ag | | | | 0.04 | | | 0.05 | | | 0.05 | | | 0.05 | | | 0.08 | | | 0.06 | | | 0.07 | | | 0.05 | | 0.06 | | | 0.06 | | | 0.05 | | | 0.06 | | | 0.06 | | | 0.06 | | | 0.06 | | 0.06 | | | 0.05 | | |
| Al | | | | 1 | | | 1 | | | 1 | | | 1 | | | 1 | | | 1 | | | 1 | | | 1 | | 1 | | | 1 | | | 1 | | | 1 | | | 1 | | | 1 | | | 1 | | 1 | | | 1 | | |
| As | | | | 9.68 | | | 9.46 | | | 10.04 | | | 9.33 | | | 9.11 | | | 5.23 | | | 7.27 | | | 8.26 | | 9.68 | | | 8.14 | | | 8.18 | | | 8.76 | | | 7.76 | | | 6.65 | | | 7.53 | | 8.63 | | | 12.52 | | |
| Ba | | | | 0.65 | | | 0.52 | | | 0.62 | | | 0.57 | | | 0.58 | | | 0.83 | | | 0.78 | | | 0.48 | | 0.34 | | | 0.65 | | | 0.93 | | | 0.43 | | | 0.64 | | | 0.71 | | | 0.6 | | 0.69 | | | 0.08 | | |
| Be | | | | 0.36 | | | 0.32 | | | 0.31 | | | 0.29 | | | 0.29 | | | 0.25 | | | 0.28 | | | 0.3 | | 0.25 | | | 0.33 | | | 0.39 | | | 0.28 | | | 0.32 | | | 0.34 | | | 0.29 | | 0.35 | | | 0.19 | | |
| Cd | | | | 2.69 | | | 3.25 | | | 4.95 | | | 3.94 | | | 2.64 | | | 7.71 | | | 4.69 | | | 2 | | 2.19 | | | 4.98 | | | 4.69 | | | 3.17 | | | 4.37 | | | 4.78 | | | 4.47 | | 4.71 | | | 0.85 | | |
| Co | | | | 0.68 | | | 0.99 | | | 0.87 | | | 1.01 | | | 0.87 | | | 0.76 | | | 0.73 | | | 0.99 | | 0.83 | | | 0.93 | | | 0.94 | | | 0.92 | | | 0.9 | | | 0.88 | | | 0.81 | | 0.9 | | | 0.73 | | |
| Cr | | | | 1.01 | | | 2.3 | | | 1.93 | | | 2.37 | | | 1.35 | | | 1.24 | | | 1.11 | | | 1.88 | | 1.68 | | | 2.12 | | | 1.95 | | | 1.82 | | | 2.05 | | | 2.05 | | | 1.81 | | 1.74 | | | 2.15 | | |
| Cu | | | | 0.34 | | | 0.29 | | | 0.29 | | | 0.29 | | | 0.28 | | | 0.2 | | | 0.33 | | | 0.21 | | 0.18 | | | 0.3 | | | 0.31 | | | 0.22 | | | 0.27 | | | 0.3 | | | 0.27 | | 0.22 | | | 0.02 | | |
| Fe | | | | 2.2 | | | 1.81 | | | 2.16 | | | 2.05 | | | 1.35 | | | 2.67 | | | 1.97 | | | 1.71 | | 1.39 | | | 2.42 | | | 2.84 | | | 1.66 | | | 2.16 | | | 2.36 | | | 2.24 | | 2.47 | | | 0.72 | | |
| Li | | | | 1.82 | | | 1.45 | | | 1.57 | | | 1.39 | | | 1.45 | | | 1.43 | | | 1.52 | | | 1.25 | | 1.21 | | | 1.59 | | | 1.48 | | | 1.26 | | | 1.49 | | | 1.68 | | | 1.42 | | 1.54 | | | 0.84 | | |
| Mg | | | | 3.87 | | | 4.22 | | | 3.9 | | | 4.02 | | | 2.14 | | | 1.82 | | | 2.2 | | | 2.67 | | 2.79 | | | 3.81 | | | 3.71 | | | 3.09 | | | 3.01 | | | 3.23 | | | 3.09 | | 2.88 | | | 1.74 | | |
| Mn | | | | 0.82 | | | 0.81 | | | 0.86 | | | 0.78 | | | 0.77 | | | 0.58 | | | 0.73 | | | 0.8 | | 0.61 | | | 0.89 | | | 0.95 | | | 0.73 | | | 0.81 | | | 0.93 | | | 0.8 | | 1.06 | | | 0.45 | | |
| Ni | | | | 1.43 | | | 3.36 | | | 2.74 | | | 3.49 | | | 2.62 | | | 2 | | | 2.05 | | | 2.79 | | 2.51 | | | 3.2 | | | 2.73 | | | 2.83 | | | 3.14 | | | 3.1 | | | 2.76 | | 2.61 | | | 1.87 | | |
| Pb | | | | 2.59 | | | 2.01 | | | 2.57 | | | 2.49 | | | 2.43 | | | 3.28 | | | 3.05 | | | 2.2 | | 1.69 | | | 3.05 | | | 4.05 | | | 2.08 | | | 2.74 | | | 3.12 | | | 2.77 | | 3.59 | | | 1.05 | | |
| Sb | | | | 0.31 | | | 0.29 | | | 0.28 | | | 0.26 | | | 0.26 | | | 0.61 | | | 0.89 | | | 0.36 | | 0.44 | | | 0.38 | | | 0.39 | | | 0.25 | | | 0.26 | | | 0.28 | | | 0.43 | | 0.42 | | | 0.3 | | |
| Se | | | | 168 | | | 117 | | | 159 | | | 162 | | | 99 | | | 159 | | | 146 | | | 63 | | 89 | | | 115 | | | 131 | | | 81 | | | 154 | | | 182 | | | 170 | | 161 | | | 48 | | |
| Sn | | | | 1.22 | | | 1.16 | | | 0.46 | | | 0.77 | | | 2.23 | | | 0.36 | | | 0.75 | | | 1.23 | | 1.32 | | | 1.11 | | | 1.24 | | | 1.07 | | | 0.58 | | | 0.82 | | | 0.58 | | 0.67 | | | 2.04 | | |
| Sr | | | | 0.36 | | | 0.8 | | | 1 | | | 1 | | | 0.42 | | | 0.37 | | | 0.51 | | | 0.48 | | 0.55 | | | 1.57 | | | 1.28 | | | 0.71 | | | 0.94 | | | 1.07 | | | 1.05 | | 0.81 | | | 0.27 | | |
| Tl | | | | 1.07 | | | 0.99 | | | 1.38 | | | 1.14 | | | 1.05 | | | 1.67 | | | 1.47 | | | 1.06 | | 0.82 | | | 1.65 | | | 1.74 | | | 1.05 | | | 1.54 | | | 1.71 | | | 1.49 | | 1.41 | | | 0.51 | | |
| V | | | | 0.51 | | | 0.49 | | | 0.51 | | | 0.47 | | | 0.42 | | | 0.54 | | | 0.47 | | | 0.43 | | 0.36 | | | 0.51 | | | 0.57 | | | 0.42 | | | 0.48 | | | 0.52 | | | 0.44 | | 0.47 | | | 0.39 | | |
| Zn | | | | 3.46 | | | 2.79 | | | 3.28 | | | 3.01 | | | 2.49 | | | 4.14 | | | 3.69 | | | 2.64 | | 2.21 | | | 3.92 | | | 4.6 | | | 2.72 | | | 3.3 | | | 3.8 | | | 3.47 | | 3.6 | | | 1.13 | | |
|  |  |  |  | |  |  | |  |  | |  |  | |  |  | |  |  | |  |  | |  |  | |  | |  |  | |  |  | |  |  | |  |  | |  |  | |  |  | |  | |  |  | |  |  |

**Table S4**. Geo-accumulation index (Igeo) for the metal(loid)s from Usumacinta River sediments in the middle (M) and lower (L) basins during the dry (up) and rainy (down) seasons.

| Metal(loid) | M1 | M3 | M4 | M5 | M6 | M7 | M8 | M9 | M10 | L1 | L2 | L3 | L4 | L5 | L7 | L9 | L10 |
| --- | --- | --- | --- | --- | --- | --- | --- | --- | --- | --- | --- | --- | --- | --- | --- | --- | --- |
| (µg g^-1^) |  |  |  |  |  |  |  |  |  |  |  |  |  |  |  |  |  |
| Ag | -0.23 | -0.21 | -0.23 | -0.25 | -0.28 | -0.21 | -0.23 | -0.26 | -0.21 | -0.18 | -0.22 | -0.18 | -0.13 | -0.12 | -0.19 | -0.14 | -0.2 |
| Al | 0 | 0 | 0 | 0 | 0 | 0 | 0 | 0 | 0 | 0 | 0 | 0 | 0 | 0 | 0 | 0 | 0 |
| As | 0.55 | 0.69 | 0.15 | 0.02 | 0.13 | 0.45 | 0.26 | 0.17 | 0.29 | 0.41 | 0.27 | 0.39 | 0.61 | 0.7 | 0.2 | 0.82 | -0.01 |
| Ba | 0.01 | 0.01 | 0.01 | 0.01 | 0.01 | 0.01 | 0.01 | 0.01 | 0.01 | 0.01 | 0.01 | 0.01 | 0.01 | 0.01 | 0.01 | 0.01 | 0.01 |
| Be | -0.41 | -0.45 | -0.67 | -0.76 | -0.65 | -0.64 | -0.54 | -0.61 | -0.58 | -0.48 | -0.65 | -0.47 | -0.44 | -0.43 | -0.55 | -0.37 | -0.62 |
| Cd | -4.95 | -4.04 | -3.68 | -5.68 | -6.05 | -3.09 | -4.95 | -5.56 | -3.84 | -2.88 | -3.93 | -2.87 | -1.17 | -1.09 | -3.44 | -1.64 | -3.47 |
| Co | 0.06 | 0.08 | 0.07 | 0.09 | 0.07 | 0.07 | 0.07 | 0.07 | 0.07 | 0.08 | 0.06 | 0.08 | 0.08 | 0.08 | 0.07 | 0.07 | 0.06 |
| Cr | 0.03 | 0.04 | 0.04 | 0.05 | 0.04 | 0.04 | 0.04 | 0.04 | 0.04 | 0.04 | 0.04 | 0.04 | 0.04 | 0.04 | 0.04 | 0.04 | 0.04 |
| Cu | 0.03 | 0.03 | 0.01 | 0.01 | 0.01 | 0.01 | 0.02 | 0.02 | 0.02 | 0.03 | 0.01 | 0.03 | 0.03 | 0.03 | 0.02 | 0.03 | 0.01 |
| Fe | 0 | 0 | 0 | 0 | 0 | 0 | 0 | 0 | 0 | 0 | 0 | 0 | 0 | 0 | 0 | 0 | 0 |
| Li | 0.1 | 0.1 | 0.05 | 0.05 | 0.07 | 0.07 | 0.09 | 0.07 | 0.09 | 0.1 | 0.06 | 0.1 | 0.11 | 0.11 | 0.09 | 0.11 | 0.07 |
| Mg | 0 | 0 | 0 | 0 | 0 | 0 | 0 | 0 | 0 | 0 | 0 | 0 | 0 | 0 | 0 | 0 | 0 |
| Mn | 0.01 | 0.01 | 0 | 0.01 | 0.01 | 0.01 | 0 | 0.01 | 0.01 | 0.01 | 0 | 0.01 | 0.01 | 0.01 | 0 | 0.01 | 0 |
| Ni | 0.04 | 0.06 | 0.05 | 0.07 | 0.06 | 0.05 | 0.05 | 0.05 | 0.05 | 0.05 | 0.05 | 0.05 | 0.06 | 0.06 | 0.05 | 0.05 | 0.04 |
| Pb | 0.12 | 0.16 | 0.08 | 0.07 | 0.11 | 0.11 | 0.14 | 0.11 | 0.1 | 0.14 | 0.09 | 0.14 | 0.17 | 0.17 | 0.1 | 0.17 | 0.1 |
| Sb | -5.55 | -6.52 | -5.56 | -7.28 | -7.45 | -4.82 | -6.6 | -6.48 | -4.75 | -4.46 | -4.73 | -4.63 | -3.16 | -2.95 | -4.31 | -2.96 | -4.09 |
| Se | -14.52 | -3.44 | -20.27 | -25.08 | -37.13 | -28.17 | -21.11 | -12.47 | -33.34 | -11.55 | -34.49 | -16.19 | -8.03 | -1.8 | -15.39 | -11.53 | -17.48 |
| Sn | 0.26 | -0.08 | 0.07 | -0.02 | 0.15 | -0.21 | 0.01 | 0.11 | 0.1 | -0.05 | 0.14 | 0.03 | -0.01 | -0.07 | 0.17 | 0.19 | 0.12 |
| Sr | 0.01 | 0.01 | 0.01 | 0.01 | 0.01 | 0.01 | 0.01 | 0.01 | 0.01 | 0.01 | 0.01 | 0.01 | 0.01 | 0.01 | 0.01 | 0.01 | 0.01 |
| Tl | -4.54 | -4.05 | -5.33 | -5.22 | -6.47 | -5.3 | -5.14 | -6.64 | -5.91 | -4.4 | -7.12 | -4.63 | -3.77 | -3.64 | -5.47 | -4.55 | -6.46 |
| V | 0.02 | 0.02 | 0.02 | 0.02 | 0.01 | 0.02 | 0.02 | 0.02 | 0.02 | 0.02 | 0.01 | 0.02 | 0.02 | 0.02 | 0.02 | 0.02 | 0.02 |
| Zn | 0.05 | 0.06 | 0.05 | 0.05 | 0.05 | 0.05 | 0.05 | 0.05 | 0.05 | 0.05 | 0.05 | 0.06 | 0.06 | 0.06 | 0.05 | 0.06 | 0.05 |

| Metal(loid) | M1 | M3 | M4 | M5 | M6 | M7 | M8 | M9 | M10 | L1 | L2 | L3 | L4 | L5 | L7 | L9 | L10 |
| --- | --- | --- | --- | --- | --- | --- | --- | --- | --- | --- | --- | --- | --- | --- | --- | --- | --- |
| (µg g^-1^) |  |  |  |  |  |  |  |  |  |  |  |  |  |  |  |  |  |
| Ag | -0.36 | -0.37 | -0.32 | -0.33 | -0.35 | -0.23 | -0.27 | -0.36 | -0.39 | -0.28 | -0.29 | -0.34 | -0.29 | -0.27 | -0.28 | -0.27 | -0.52 |
| Al | 0 | 0 | 0 | 0 | 0 | 0 | 0 | 0 | 0 | 0 | 0 | 0 | 0 | 0 | 0 | 0 | 0 |
| As | 0.78 | 0.66 | 0.85 | 0.75 | 0.48 | 0.73 | 0.67 | 0.58 | 0.47 | 0.84 | 0.92 | 0.56 | 0.72 | 0.76 | 0.77 | 0.86 | 0.17 |
| Ba | 0.01 | 0.01 | 0.01 | 0.01 | 0.01 | 0.01 | 0.01 | 0.01 | 0.01 | 0.01 | 0.01 | 0.01 | 0.01 | 0.01 | 0.01 | 0.01 | 0 |
| Be | -0.48 | -0.52 | -0.53 | -0.55 | -0.56 | -0.6 | -0.57 | -0.54 | -0.6 | -0.5 | -0.45 | -0.56 | -0.52 | -0.5 | -0.56 | -0.49 | -0.7 |
| Cd | -9.72 | -8.81 | -6.78 | -7.88 | -9.8 | -4.65 | -7.04 | -11.13 | -10.69 | -6.75 | -7.03 | -8.93 | -7.37 | -6.94 | -7.27 | -7.02 | -15.23 |
| Co | 0.06 | 0.07 | 0.06 | 0.07 | 0.06 | 0.06 | 0.06 | 0.07 | 0.06 | 0.07 | 0.07 | 0.07 | 0.07 | 0.07 | 0.06 | 0.07 | 0.06 |
| Cr | 0.03 | 0.04 | 0.04 | 0.04 | 0.03 | 0.03 | 0.03 | 0.04 | 0.04 | 0.04 | 0.04 | 0.04 | 0.04 | 0.04 | 0.04 | 0.04 | 0.04 |
| Cu | 0.03 | 0.02 | 0.02 | 0.02 | 0.02 | 0.02 | 0.03 | 0.02 | 0.02 | 0.02 | 0.03 | 0.02 | 0.02 | 0.02 | 0.02 | 0.02 | -0.02 |
| Fe | 0 | 0 | 0 | 0 | 0 | 0 | 0 | 0 | 0 | 0 | 0 | 0 | 0 | 0 | 0 | 0 | 0 |
| Li | 0.11 | 0.09 | 0.1 | 0.09 | 0.09 | 0.09 | 0.1 | 0.09 | 0.09 | 0.1 | 0.1 | 0.09 | 0.1 | 0.1 | 0.09 | 0.1 | 0.07 |
| Mg | 0 | 0 | 0 | 0 | 0 | 0 | 0 | 0 | 0 | 0 | 0 | 0 | 0 | 0 | 0 | 0 | 0 |
| Mn | 0.01 | 0.01 | 0.01 | 0.01 | 0.01 | 0 | 0.01 | 0.01 | 0.01 | 0.01 | 0.01 | 0.01 | 0.01 | 0.01 | 0.01 | 0.01 | 0 |
| Ni | 0.04 | 0.05 | 0.05 | 0.05 | 0.05 | 0.05 | 0.05 | 0.05 | 0.05 | 0.05 | 0.05 | 0.05 | 0.05 | 0.05 | 0.05 | 0.05 | 0.05 |
| Pb | 0.16 | 0.14 | 0.16 | 0.16 | 0.15 | 0.18 | 0.17 | 0.15 | 0.13 | 0.17 | 0.19 | 0.14 | 0.16 | 0.17 | 0.17 | 0.19 | 0.09 |
| Sb | -20.05 | -20.39 | -20.61 | -20.92 | -20.89 | -16.83 | -15.05 | -19.44 | -18.41 | -19.13 | -19.04 | -21.08 | -20.99 | -20.63 | -18.53 | -18.7 | -20.32 |
| Se | 14.04 | 7.12 | 12.93 | 13.35 | 3.9 | 13.02 | 11.25 | -4.9 | 1.89 | 6.75 | 9.23 | 0.08 | 12.41 | 15.54 | 14.22 | 13.2 | -10.26 |
| Sn | -0.24 | -0.27 | -0.71 | -0.47 | 0.05 | -0.84 | -0.48 | -0.24 | -0.21 | -0.29 | -0.23 | -0.31 | -0.6 | -0.43 | -0.6 | -0.53 | 0 |
| Sr | 0.01 | 0.01 | 0.01 | 0.01 | 0.01 | 0.01 | 0.01 | 0.01 | 0.01 | 0.01 | 0.01 | 0.01 | 0.01 | 0.01 | 0.01 | 0.01 | 0.01 |
| Tl | -4.54 | -4.71 | -4 | -4.41 | -4.59 | -3.6 | -3.88 | -4.57 | -5.13 | -3.63 | -3.52 | -4.59 | -3.78 | -3.55 | -3.85 | -3.97 | -6.15 |
| V | 0.02 | 0.02 | 0.02 | 0.02 | 0.02 | 0.02 | 0.02 | 0.02 | 0.02 | 0.02 | 0.02 | 0.02 | 0.02 | 0.02 | 0.02 | 0.02 | 0.02 |
| Zn | 0.06 | 0.05 | 0.06 | 0.05 | 0.05 | 0.06 | 0.06 | 0.05 | 0.05 | 0.06 | 0.06 | 0.05 | 0.06 | 0.06 | 0.06 | 0.06 | 0.04 |

**Table S5**. Contamination factor (CF) for metal(loid)s from Usumacinta River sediments in the middle (M) and lower (L) basins during the dry (up) and rainy (down) seasons.

| Metal(loid) | M1 | M3 | M4 | M5 | M6 | M7 | M8 | M9 | M10 | L1 | L2 | L3 | L4 | L5 | L7 | L9 | L10 |
| --- | --- | --- | --- | --- | --- | --- | --- | --- | --- | --- | --- | --- | --- | --- | --- | --- | --- |
| (µg g^-1^) |  |  |  |  |  |  |  |  |  |  |  |  |  |  |  |  |  |
| Ag | 0.03 | 0.03 | 0.03 | 0.02 | 0.02 | 0.03 | 0.03 | 0.02 | 0.03 | 0.04 | 0.03 | 0.04 | 0.05 | 0.06 | 0.04 | 0.05 | 0.03 |
| Al | 0.13 | 0.27 | 0.07 | 0.18 | 0.05 | 0.11 | 0.13 | 0.06 | 0.08 | 0.17 | 0.04 | 0.16 | 0.28 | 0.33 | 0.1 | 0.22 | 0.05 |
| As | 1.55 | 2.01 | 0.73 | 0.58 | 0.71 | 1.29 | 0.91 | 0.76 | 0.96 | 1.19 | 0.93 | 1.15 | 1.73 | 2.05 | 0.81 | 2.56 | 0.55 |
| Ba | 0.07 | 0.15 | 0.02 | 0.05 | 0.08 | 0.06 | 0.1 | 0.04 | 0.04 | 0.07 | 0.04 | 0.09 | 0.15 | 0.16 | 0.04 | 0.24 | 0.03 |
| Be | 0.11 | 0.1 | 0.05 | 0.04 | 0.05 | 0.06 | 0.07 | 0.06 | 0.07 | 0.09 | 0.05 | 0.09 | 0.1 | 0.1 | 0.07 | 0.12 | 0.06 |
| Cd | 1.79 | 2.16 | 2.33 | 1.53 | 1.42 | 2.63 | 1.79 | 1.57 | 2.25 | 2.75 | 2.21 | 2.75 | 3.92 | 3.98 | 2.45 | 3.56 | 2.43 |
| Co | 0.18 | 0.35 | 0.23 | 0.4 | 0.24 | 0.27 | 0.26 | 0.22 | 0.23 | 0.29 | 0.18 | 0.29 | 0.29 | 0.28 | 0.27 | 0.27 | 0.2 |
| Cr | 0.21 | 0.83 | 0.79 | 1.5 | 0.61 | 0.62 | 0.43 | 0.61 | 0.51 | 0.52 | 0.47 | 0.53 | 0.56 | 0.55 | 0.44 | 0.43 | 0.56 |
| Cu | 0.11 | 0.1 | 0.03 | 0.03 | 0.04 | 0.04 | 0.07 | 0.05 | 0.06 | 0.08 | 0.03 | 0.08 | 0.11 | 0.11 | 0.05 | 0.1 | 0.03 |
| Fe | 0.42 | 0.66 | 0.18 | 0.81 | 0.22 | 0.42 | 0.38 | 0.35 | 0.34 | 0.46 | 0.19 | 0.45 | 0.65 | 0.73 | 0.32 | 0.62 | 0.22 |
| Li | 0.44 | 0.39 | 0.15 | 0.14 | 0.21 | 0.21 | 0.35 | 0.23 | 0.3 | 0.41 | 0.17 | 0.4 | 0.46 | 0.46 | 0.35 | 0.51 | 0.2 |
| Mg | 0.59 | 1.08 | 0.64 | 2.18 | 0.65 | 0.51 | 0.57 | 0.54 | 0.62 | 0.74 | 0.35 | 0.74 | 0.8 | 0.85 | 0.63 | 0.64 | 0.41 |
| Mn | 0.16 | 0.23 | 0.12 | 0.21 | 0.42 | 0.17 | 0.15 | 0.17 | 0.15 | 0.17 | 0.13 | 0.18 | 0.2 | 0.21 | 0.14 | 0.25 | 0.11 |
| Ni | 0.25 | 1.43 | 0.69 | 2.64 | 1.05 | 0.72 | 0.73 | 0.6 | 0.64 | 0.89 | 0.69 | 0.93 | 1.01 | 0.98 | 0.71 | 0.73 | 0.44 |
| Pb | 0.38 | 0.61 | 0.22 | 0.21 | 0.32 | 0.33 | 0.47 | 0.32 | 0.31 | 0.49 | 0.26 | 0.51 | 0.69 | 0.76 | 0.29 | 0.76 | 0.3 |
| Sb | 1.58 | 1.29 | 1.57 | 1.1 | 1.06 | 1.83 | 1.27 | 1.3 | 1.86 | 1.98 | 1.87 | 1.91 | 2.59 | 2.71 | 2.04 | 2.7 | 2.13 |
| Se | 9.4 | 16.73 | 6.97 | 5.43 | 2.9 | 4.62 | 6.68 | 10.46 | 3.53 | 10.97 | 3.33 | 8.62 | 13.17 | 18.21 | 8.99 | 10.98 | 8.06 |
| Sn | 0.86 | 0.42 | 0.58 | 0.47 | 0.68 | 0.32 | 0.51 | 0.63 | 0.62 | 0.45 | 0.66 | 0.54 | 0.49 | 0.43 | 0.71 | 0.74 | 0.65 |
| Sr | 0.06 | 0.34 | 0.2 | 0.25 | 0.09 | 0.15 | 0.13 | 0.1 | 0.15 | 0.48 | 0.06 | 0.21 | 0.28 | 0.31 | 0.12 | 0.2 | 0.08 |
| Tl | 0.27 | 0.33 | 0.18 | 0.19 | 0.11 | 0.19 | 0.2 | 0.1 | 0.14 | 0.28 | 0.08 | 0.25 | 0.38 | 0.4 | 0.17 | 0.26 | 0.11 |
| V | 0.12 | 0.14 | 0.1 | 0.11 | 0.06 | 0.12 | 0.12 | 0.16 | 0.15 | 0.11 | 0.05 | 0.11 | 0.13 | 0.13 | 0.1 | 0.13 | 0.11 |
| Zn | 0.73 | 0.94 | 0.42 | 0.71 | 0.49 | 0.74 | 0.7 | 0.55 | 0.67 | 0.76 | 0.39 | 0.95 | 1.1 | 1.12 | 0.65 | 1 | 0.5 |

| Metal(loid) | M1 | M3 | M4 | M5 | M6 | M7 | M8 | M9 | M10 | L1 | L2 | L3 | L4 | L5 | L7 | L9 | L10 |
| --- | --- | --- | --- | --- | --- | --- | --- | --- | --- | --- | --- | --- | --- | --- | --- | --- | --- |
| (µg g^-1^) |  |  |  |  |  |  |  |  |  |  |  |  |  |  |  |  |  |
| Ag | 0.01 | 0.01 | 0.01 | 0.01 | 0.01 | 0.03 | 0.02 | 0.01 | 0.01 | 0.02 | 0.02 | 0.01 | 0.02 | 0.02 | 0.02 | 0.02 | 0.01 |
| Al | 0.25 | 0.2 | 0.27 | 0.24 | 0.15 | 0.42 | 0.27 | 0.2 | 0.14 | 0.33 | 0.38 | 0.18 | 0.27 | 0.35 | 0.31 | 0.32 | 0.06 |
| As | 2.39 | 1.92 | 2.72 | 2.25 | 1.36 | 2.18 | 1.96 | 1.64 | 1.34 | 2.65 | 3.11 | 1.58 | 2.13 | 2.31 | 2.33 | 2.77 | 0.77 |
| Ba | 0.16 | 0.13 | 0.15 | 0.14 | 0.14 | 0.2 | 0.19 | 0.12 | 0.08 | 0.16 | 0.23 | 0.11 | 0.16 | 0.18 | 0.15 | 0.17 | 0.02 |
| Be | 0.09 | 0.08 | 0.08 | 0.07 | 0.07 | 0.06 | 0.07 | 0.07 | 0.06 | 0.08 | 0.1 | 0.07 | 0.08 | 0.08 | 0.07 | 0.09 | 0.05 |
| Cd | 0.66 | 0.8 | 1.22 | 0.97 | 0.65 | 1.9 | 1.16 | 0.49 | 0.54 | 1.23 | 1.16 | 0.78 | 1.08 | 1.18 | 1.1 | 1.16 | 0.21 |
| Co | 0.17 | 0.25 | 0.21 | 0.25 | 0.21 | 0.19 | 0.18 | 0.24 | 0.2 | 0.23 | 0.23 | 0.23 | 0.22 | 0.22 | 0.2 | 0.22 | 0.18 |
| Cr | 0.25 | 0.57 | 0.48 | 0.58 | 0.33 | 0.31 | 0.27 | 0.46 | 0.42 | 0.52 | 0.48 | 0.45 | 0.51 | 0.51 | 0.45 | 0.43 | 0.53 |
| Cu | 0.08 | 0.07 | 0.07 | 0.07 | 0.07 | 0.05 | 0.08 | 0.05 | 0.04 | 0.07 | 0.08 | 0.05 | 0.07 | 0.07 | 0.07 | 0.05 | 0.01 |
| Fe | 0.54 | 0.45 | 0.53 | 0.51 | 0.33 | 0.66 | 0.49 | 0.42 | 0.34 | 0.6 | 0.7 | 0.41 | 0.53 | 0.58 | 0.55 | 0.61 | 0.18 |
| Li | 0.45 | 0.36 | 0.39 | 0.34 | 0.36 | 0.35 | 0.37 | 0.31 | 0.3 | 0.39 | 0.37 | 0.31 | 0.37 | 0.42 | 0.35 | 0.38 | 0.21 |
| Mg | 0.95 | 1.04 | 0.96 | 0.99 | 0.53 | 0.45 | 0.54 | 0.66 | 0.69 | 0.94 | 0.92 | 0.76 | 0.74 | 0.8 | 0.76 | 0.71 | 0.43 |
| Mn | 0.2 | 0.2 | 0.21 | 0.19 | 0.19 | 0.14 | 0.18 | 0.2 | 0.15 | 0.22 | 0.23 | 0.18 | 0.2 | 0.23 | 0.2 | 0.26 | 0.11 |
| Ni | 0.35 | 0.83 | 0.68 | 0.86 | 0.65 | 0.49 | 0.51 | 0.69 | 0.62 | 0.79 | 0.67 | 0.7 | 0.77 | 0.77 | 0.68 | 0.64 | 0.46 |
| Pb | 0.64 | 0.5 | 0.63 | 0.61 | 0.6 | 0.81 | 0.75 | 0.54 | 0.42 | 0.75 | 1 | 0.51 | 0.68 | 0.77 | 0.68 | 0.89 | 0.26 |
| Sb | 0.08 | 0.07 | 0.07 | 0.06 | 0.06 | 0.15 | 0.22 | 0.09 | 0.11 | 0.09 | 0.1 | 0.06 | 0.06 | 0.07 | 0.11 | 0.1 | 0.07 |
| Se | 41.5 | 28.96 | 39.16 | 40.04 | 24.5 | 39.36 | 35.9 | 15.5 | 22.06 | 28.4 | 32.32 | 20.08 | 38.12 | 44.86 | 41.88 | 39.72 | 11.73 |
| Sn | 0.3 | 0.29 | 0.11 | 0.19 | 0.55 | 0.09 | 0.19 | 0.3 | 0.32 | 0.27 | 0.31 | 0.26 | 0.14 | 0.2 | 0.14 | 0.17 | 0.5 |
| Sr | 0.09 | 0.2 | 0.25 | 0.25 | 0.1 | 0.09 | 0.13 | 0.12 | 0.14 | 0.39 | 0.32 | 0.17 | 0.23 | 0.26 | 0.26 | 0.2 | 0.07 |
| Tl | 0.27 | 0.25 | 0.34 | 0.28 | 0.26 | 0.41 | 0.36 | 0.26 | 0.2 | 0.41 | 0.43 | 0.26 | 0.38 | 0.42 | 0.37 | 0.35 | 0.12 |
| V | 0.13 | 0.12 | 0.13 | 0.12 | 0.1 | 0.13 | 0.12 | 0.11 | 0.09 | 0.13 | 0.14 | 0.1 | 0.12 | 0.13 | 0.11 | 0.12 | 0.1 |
| Zn | 0.85 | 0.69 | 0.81 | 0.74 | 0.61 | 1.02 | 0.91 | 0.65 | 0.55 | 0.97 | 1.13 | 0.67 | 0.82 | 0.94 | 0.86 | 0.89 | 0.28 |

**Table S6**. The pollution load index (PLI) for the metal(loid)s in the different sampling stations from Usumacinta River sediments in the middle (M) and lower (L) basins during the dry (up) and rainy (down) seasons.

| M1 | M3 | M4 | M5 | M6 | M7 | M8 | M9 | M10 | L1 | L2 | L3 | L4 | L5 | L7 | L9 | L10 |
| --- | --- | --- | --- | --- | --- | --- | --- | --- | --- | --- | --- | --- | --- | --- | --- | --- |
| 0.315 | 0.485 | 0.253 | 0.336 | 0.25 | 0.298 | 0.319 | 0.269 | 0.289 | 0.403 | 0.21 | 0.391 | 0.500 | 0.527 | 0.304 | 0.491 | 0.234 |
|  | | | | | | | | | | | | | | | | |
| M1 | M3 | M4 | M5 | M6 | M7 | M8 | M9 | M10 | L1 | L2 | L3 | L4 | L5 | L7 | L9 | L10 |
| 0.307 | 0.317 | 0.34 | 0.335 | 0.276 | 0.330 | 0.332 | 0.275 | 0.248 | 0.389 | 0.413 | 0.280 | 0.339 | 0.377 | 0.345 | 0.362 | 0.144 |
